# The Arabidopsis SUMO E3 ligase SIZ1 mediates the temperature dependent trade-off between plant immunity and growth

**DOI:** 10.1371/journal.pgen.1007157

**Published:** 2018-01-22

**Authors:** Valentin Hammoudi, Like Fokkens, Bas Beerens, Georgios Vlachakis, Sayantani Chatterjee, Manuel Arroyo-Mateos, Paul F. K. Wackers, Martijs J. Jonker, Harrold A. van den Burg

**Affiliations:** 1 Molecular Plant Pathology, University of Amsterdam, Amsterdam, The Netherlands; 2 RNA Biology and Applied Bioinformatics, University of Amsterdam, Amsterdam, The Netherlands; Martin-Luther-University Halle-Wittenberg, GERMANY

## Abstract

Increased ambient temperature is inhibitory to plant immunity including auto-immunity. SNC1-dependent auto-immunity is, for example, fully suppressed at 28°C. We found that the Arabidopsis sumoylation mutant *siz1* displays SNC1-dependent auto-immunity at 22°C but also at 28°C, which was EDS1 dependent at both temperatures. This *siz1* auto-immune phenotype provided enhanced resistance to *Pseudomonas* at both temperatures. Moreover, the rosette size of *siz1* recovered only weakly at 28°C, while this temperature fully rescues the growth defects of other SNC1-dependent auto-immune mutants. This thermo-insensitivity of *siz1* correlated with a compromised thermosensory growth response, which was independent of the immune regulators PAD4 or SNC1. Our data reveal that this high temperature induced growth response strongly depends on COP1, while SIZ1 controls the amplitude of this growth response. This latter notion is supported by transcriptomics data, *i*.*e*. SIZ1 controls the amplitude and timing of high temperature transcriptional changes including a subset of the PIF4/BZR1 gene targets. Combined our data signify that SIZ1 suppresses an SNC1-dependent resistance response at both normal and high temperatures. At the same time, SIZ1 amplifies the dark and high temperature growth response, likely via COP1 and upstream of gene regulation by PIF4 and BRZ1.

## Author summary

Ambient temperature is a major actor in plant immunity and in growth regulation. Foremost, high temperature (>27°C) is known to block plant defence responses. High temperature also alters the plant morphology by inducing elongation growth, which facilitates plant ‘cooling’. This process is called thermomorphogenesis. Importantly, the SUMO E3 ligase SIZ1 suppresses plant immunity at normal conditions (22°C), but its role in immunity at high temperature was unknown. SIZ1 was recently shown to sumoylate and activate the ubiquitin E3 ligase COP1, a key player in thermomorphogenesis affecting the accumulation and/or stability of key transcription factors for this process (PIF4 and HY5). At high temperature PIF4 suppresses SNC1-dependent growth defects and auto-immunity for the *snc1-1* mutant. We report that part of the SNC1-dependent auto-immune response is retained and activated in the *siz1* mutant at high temperature resulting in enhanced resistance to *Pseudomonas*. In addition, we find that SIZ1 controls the thermomorphogenesis response and it affects expression of a substantial subset of PIF4 and BZR1 gene targets in response to high temperature. Our data imply that SIZ1 acts upstream of the PIF4/BZR1 hub. Combined the data highlight that SIZ1 has a dual role in the trade-off between SNC1-dependent immunity and growth at elevated temperature, where the latter aspect potentially runs via COP1.

## Introduction

Ambient temperature is a major factor that affects plant growth and development, but also plant immunity [1,2]. In particular, the temperature range of 16-32ºC modulates the output of many plant immune receptors. For example, the tobacco *N* (*Necrosis*) gene fails to trigger resistance against Tobacco mosaic virus (TMV) at 30°C, while conferring resistance at 23°C [3]. This is accompanied by the loss of the hypersensitive response (HR) above 27°C. This HR includes a localized cell death that appears to be associated with recognition of pathogen effectors resulting in effector-triggered immunity (ETI) [4–7]. Multiple examples of high temperature suppression of ETI have been described for the TNL-type of immune receptors (Toll Interleukin-1 receptor [TIR], NB-LRR-type) [2], including the tobacco immune receptor N against Tobacco mosaic virus (TMV) [7,8], but also resistance mediated by the Arabidopsis immune receptor RPS4, which recognizes the avirulence protein AvrRPS4 from *Pseudomonas*, is suppressed at high temperature [9]. Finally, SNC1 (Suppressor of *npr1-1*, constitutive 1) dependent auto-immunity in the gain-of-function mutant *snc1-1* is suppressed at high temperature [10]. Auto-immunity in the *snc1-1* mutant was caused by hyperaccumulation of a mutant variant of SNC1 resulting in a dwarf stature of the mutant plant with curly leaves at 22°C [11]; At 28°C this auto-immune phenotype of *snc1-1* is fully suppressed yielding plants with wild type rosettes without any macroscopic lesions or microscopic cell death. Importantly, HR activation by SNC1 required nuclear localization of SNC1, which appeared to be compromised when plants were kept at 28°C [6,7,12].

In non-infected plants, SNC1 levels are tightly controlled at both the transcript and protein level to prevent spurious immune signalling [13]. The expression of *SNC1* is, for example, indirectly negatively regulated by the plasma membrane-localized protein BON1 (Bonzai 1) [14], but also the protein levels of SNC1 are regulated *e*.*g*. by the immune adaptor SRFR1 (Suppressor of RPS4-RLD 1) [15,16], several protein folding chaperones [17], and the F-box protein CPR1 (Constitutive expressor of Pathogenesis-related (PR) proteins 1) [11,18]. Mutations in the corresponding genes (*e*.*g*. *snc1-1*, *bon1*, *srfr1-4* and *cpr1-2*) cause SNC1-dependent auto-immunity (hereafter SNC1^auto-I^). SNC1^auto-I^ relies on EDS1 and PAD4 (Enhanced disease susceptibility 1, Phytoalexin-deficient 4) [19]. Upon recognition of biotrophic pathogens, EDS1 translocates from the cytoplasm, where it is sequestered by the related protein PAD4, to the nucleus [20–23]. Nuclear localization of EDS1 is necessary for transcriptional reprogramming to trigger SA biosynthesis and other plant defence responses.

Strikingly, high temperature suppression of auto-immunity depends for the *snc1-1* mutant on the central growth regulator PIF4 (Phytochrome Interacting Factor 4), a transcription factor (TF) that is essential for thermomorphogenesis at 28°C [24]. This implies that plant growth is prioritized over SNC1-dependent auto-immunity at 28°C via transcriptional regulation. High ambient temperature increases PIF4 activity by controlling both its transcript levels and protein levels in a diurnal dark/light cycle [25]. This process is directly affected by relocalization of the ubiquitin E3 ligase COP1 (Constitutive Photomorphogenesis 1) to the nucleus in dark conditions. In the nucleus COP1 targets key regulators of both PIF4 protein activity and *PIF4* gene expression for degradation [26]. Recent data highlight that COP1 is not only essential for the dark-induced growth response, but also at high ambient temperature in a normal diurnal dark/light cycle [27].

Here we studied auto-immunity in a mutant of the Arabidopsis SUMO E3 ligase SIZ1. Auto-immunity of *siz1* highly resembles SNC1^auto-I^ [28,29], *i*.*e*. the mutant shows enhanced resistance to *Pseudomonas* infection due to high levels of SA, its rosette adopts a very similar morphology (including lesions and spontaneous cell death) as the SNC1^auto-I^ mutants, and this auto-immune phenotype depends on *PAD4*. Auto-immunity in the *siz1* mutant is likely caused by the absence of sumoylation on one or more of its substrates, as the *sumo1/2*^*KD*^ knock-down mutant also displays auto-immunity [29]. SIZ1 is the major SUMO E3 ligase in Arabidopsis [30], affecting SUMO conjugation of many substrates including pivotal regulators of growth [31–33]. For example, COP1 is a direct substrate of SIZ1 and its sumoylation enhances the intrinsic ubiquitin E3 ligase activity of COP1 [34,35].

As PIF4 controls the high temperature-mediated recovery of *snc1-1* auto-immunity and SIZ1 controls the activity of a key regulator of PIF4, namely COP1, we assessed here (i) whether the *siz1* auto-immune phenotype requires a functional *SNC1* gene copy at normal and high temperature. Moreover, we tested (ii) if loss of SIZ1 function suppresses the COP1/ PIF4 mediated growth response at high temperature and in dark conditions. We found that *siz1* auto-immunity is sustained at 28°C resulting in enhanced resistance to bacteria, which depended on both SNC1 and EDS1. The dwarf stature of *siz1* also hardly recovered at 28°C. Moreover, we found that *siz1* shows a compromised thermosensory growth response, which was independent of SNC1 and PAD4. This positive regulatory role of SIZ1 in growth regulation was suppressed by the TF HY5 (Elongated hypocotyl 5) at 22°C, while it depended on COP1 function at 28°C (and in dark conditions). HY5 is a direct substrate for COP1 targeted protein degradation. Finally, we found that high temperature induced transcriptome changes are both attenuated and delayed in the *siz1* and *sumo1/2*^*KD*^ mutants and that a substantial subset of the affected genes are known genomic targets for PIF4 binding and regulation.

## Results

### Hallmarks of auto-immunity are not fully suppressed at high temperature in *siz1*

**Fig 1:**
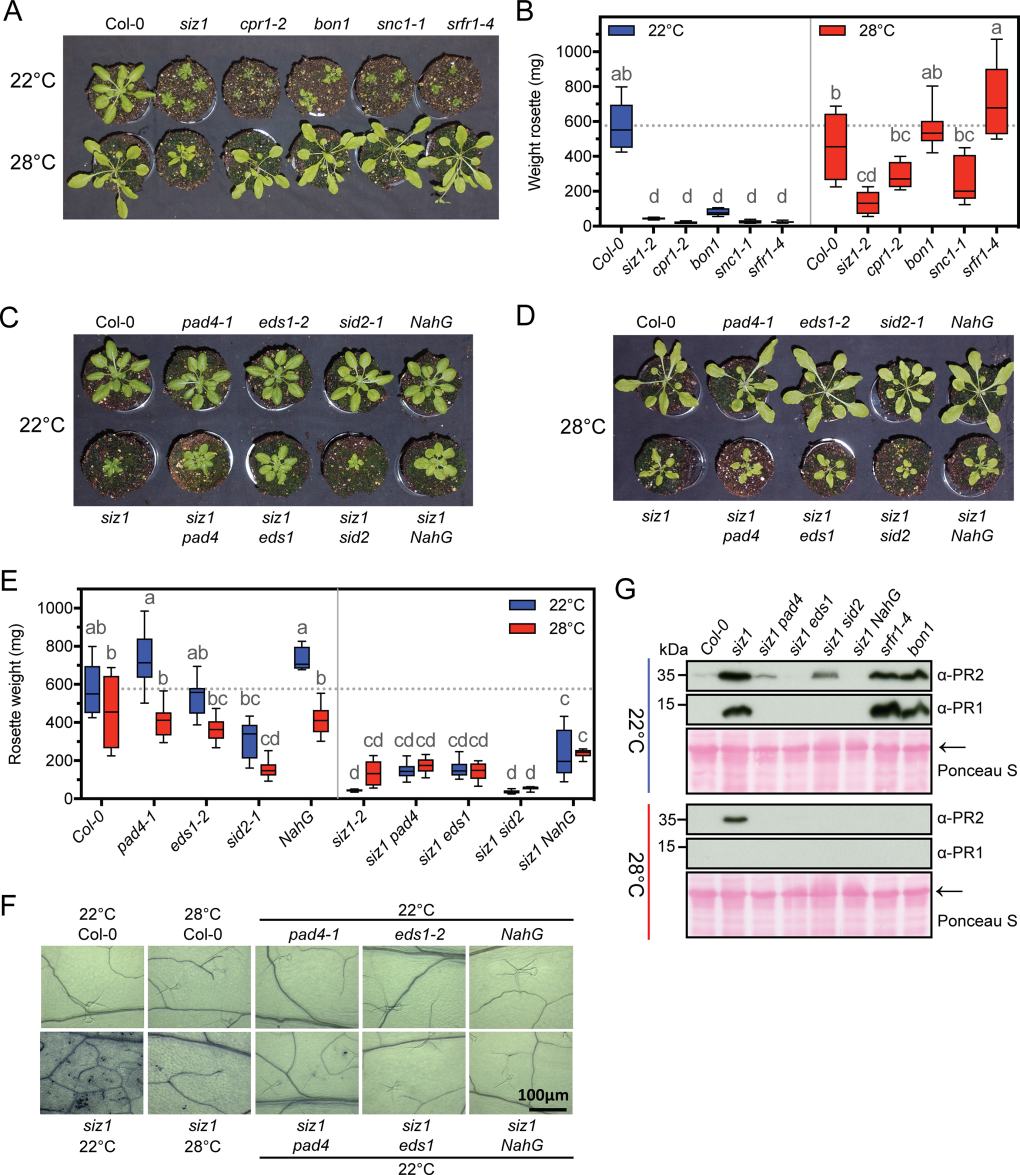
While growth retardation of *siz1* is hardly rescued at 28°C, other hallmarks of auto-immunity fully recover at this temperature. **(A)** Picture of the rosettes of *siz1* and SNC1-dependent auto-immune mutants (*crp1-2*, *bon1*, *snc1-1*, *srfr1-4*) grown for 5 weeks in SD conditions at 22°C or 28°C. The mutants are in the Col-0 background. **(B)** Box-plot (middle bar = median, box limit = upper and lower quartile, extremes = Min and Max values) depicting the rosette weight of the genotypes shown in (A). Plants were 5-week-old plants. Significant differences were detected using a two-way ANOVA with Tukey’s multiple comparisons test: Genotype *p-value*<0.0001 (29% of the variation), Temperature *p-value*<0.001 (37%); GxT interaction *p-value*<0.0001 (22%); letters indicate significantly different post hoc groups (n = 8–10). The experiment was repeated 3 times with similar result. **(C, D)** Similar to (A), plants were grown in parallel for 5-weeks at 22°C or 28°C. The top row depicts wild type Col-0 and the single mutants, while the bottom row depicts *siz1* and *siz1* crossed with the mutants of the top row. **(E)** Box-plot with the rosette weight of the plants of panels (C, D). The statistical test was similar to (B) with similar result (n = 8). *siz pad4-1*, *siz1 eds1-2*, and *siz1 NahG* show a small recovery at 22°C (C) without any additional effect at 28°C (D). The experiment was repeated three times with similar result. Left side, single mutants; right side, *siz1* and the corresponding double mutants. **(F)** Spontaneous cell death is absent in *siz1* at 28°C, but also 22°C when *EDS1*/*PAD4* are mutated or SA accumulation is compromised (*NahG*). Fully elongated leaves of 5-week-old plants were stained with Trypan blue and examined under the microscope. **(G)** Accumulation of PR1/PR2 in *siz1* double mutants grown at 22°C or 28°C. *srfr1* and *bon1* are shown as control for PR accumulation. Total protein was extracted from 5-week-old plants. PR proteins were detected with polyclonal antibodies. Blots were stained with Ponceau S to confirm equal protein loading (← = Rubisco). The blots shown are a single exposure on one film of gels run/blotted in parallel with the samples taken in parallel as well. The apparent Mw of marker proteins is shown on the left.

A hallmark of SNC1^auto-I^ is a dwarf stature and curled leaves. These morphological defects disappear when SNC1^auto-I^ mutants like *cpr1-2*, *bon1*, *snc1-1*, and *srfr1-4* are grown at 28°C, adopting a wild type stature (**Fig 1A** and **1B**). Here we tested if also for *siz1* these morphological defects are rescued when it grows at high temperature. In contrast to the four aforementioned SNC1^auto-I^ mutants, we observed that *siz1* remains significantly smaller than the wild type control at 28°C (**Fig 1A** and **Fig 1B**, compare group ‘cd’ with group b). At 22°C, the rosette weight of *siz1* was indistinguishable from these four SNC1^auto-I^ mutants (**Fig 1B,** group ‘d’). Previous work by others had shown that the auto-immune phenotype of these SNC1^auto-I^ mutants depends on (*i*) a functional gene copy of *PAD4* and *EDS1*, and (*ii*) accumulation of the defence hormone SA [10,16,36,37]. Likewise, Lee and co-workers demonstrated that the *siz1* phenotype (partially) depends on PAD4 and SA accumulation [28], but the role of EDS1 remained unknown. Since EDS1 is the major nuclear actor of the PAD4/EDS1 hub [22,38] and SIZ1 is considered to primarily act in the nucleus [39], we examined if *siz1* auto-immunity depends on EDS1. The *siz1* growth defect partially recovered when it was crossed with the *eds1-2* mutation in the Col-0 background, but this recovery did not significantly differ from the recovery seen for the double mutants *siz1 pad4* and *siz1 NahG* (a transgene encoding salicylate hydroxylase that effectively prevents SA accumulation by converting it to catechol) at 22°C (**Fig 1C** and **1E**; all post hoc group ‘c’). We also crossed *siz1* with a mutant for *SID2* (*Salicylic acid induction deficient 2*), which encodes the key enzyme for SA synthesis in plant immunity [40]. As seen by others for other auto-immune mutants [41], introduction of the *sid2* mutation did not rescue the *siz1* growth defect seen at 22°C (**Fig 1C** and **1E**, group d). Importantly, at 28°C none of the *siz1* double mutants showed any additional growth recovery compared to *siz1* alone (**Fig 1D** and **1E**, group c). This suggests that the small growth recovery of *siz1* seen at 28°C (**Fig 1E,** from only ‘d’ at 22°C to ‘cd’ at 28°C) is potentially linked to suppression of its auto-immune phenotype, which in turn would depend on EDS1/PAD4 and SA accumulation.

**Fig 2:**
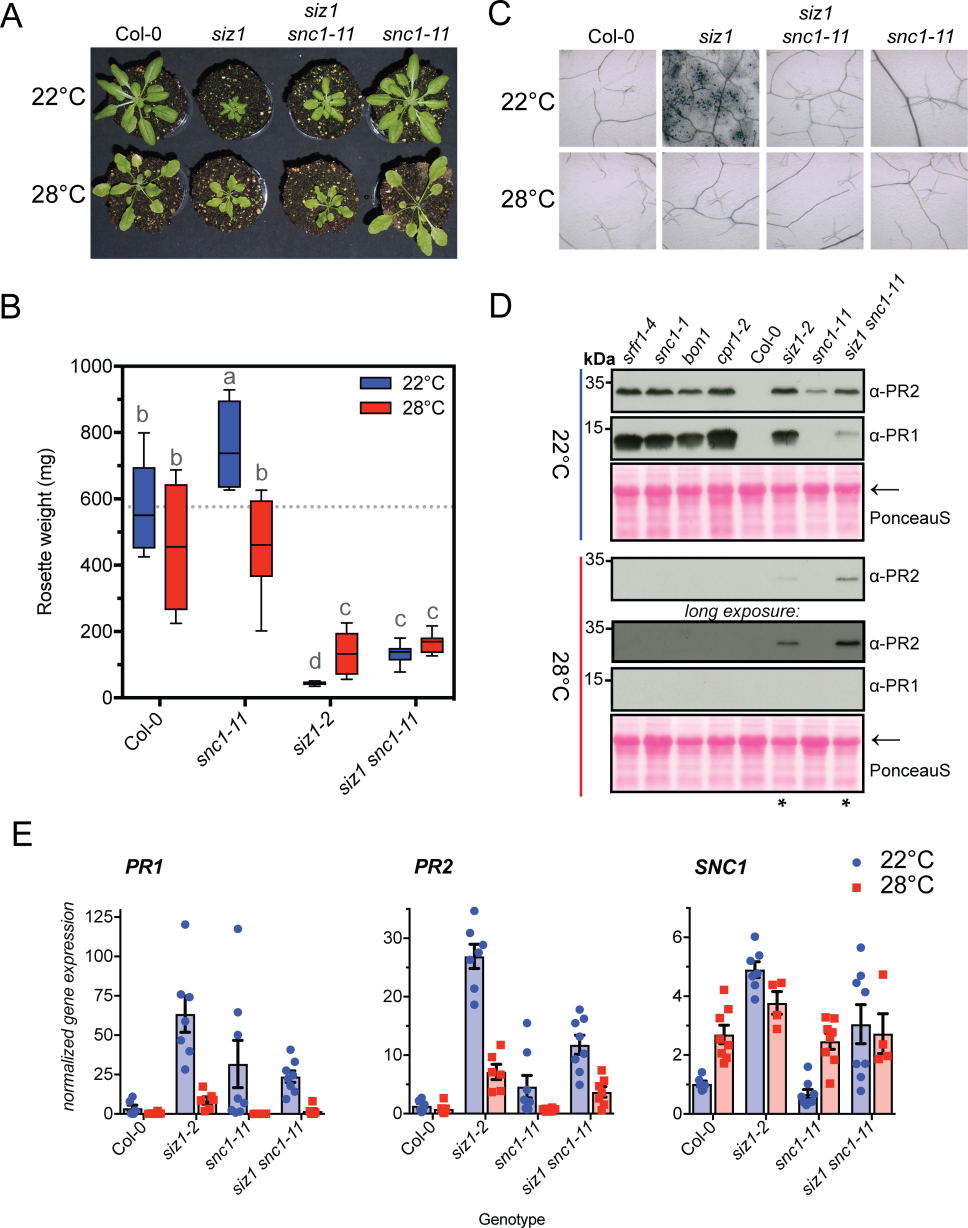
The *siz1* auto-immune phenotype is partially rescued by loss of SNC1. **(A)** Picture of the rosettes of *siz1* and the *siz1 snc1-11* double at 22°C/28°C. At 22°C growth retardation of *siz1* is partially recovered in the *snc1-11* background, while at 28°C *siz1 snc1-11* does not show any additional recovery to *siz1*. Plants were 5-weeks-old (SD). **(B)** Box plot showing the rosette weight of the plants in (A). Significant differences were determined using a two-ay ANOVA followed by a Tukey post-hoc test: Genotype *p-value*<0.0001 (75% of the variation), Temperature *p-value* = 0.0033 (2.1%); GxT interaction *p-value*<0.0001 (8.8%). The letters indicate statistically different post hoc groups (n = 8) **(C)** Spontaneous cell death in *siz1* requires SNC1 function. Fully elongated leaves of 5-week-old plants were stained with Trypan blue. **(D)** Accumulation of PR1 and PR2 is partially suppressed in *siz1 snc1-11*. As control for thermosensitive accumulation of PR proteins, *srf1-4*, *snc1-1*, *bon1* and *cpr1-2* are shown. Total protein was extracted from 5-week-old plants. The blots were prepared in parallel and ECL detection was done on one film, except for the ‘long exposure’ to reveal PR2 accumulation. Blots were stained with Ponceau S to confirm equal protein loading. Asterisks mark enhanced PR2 accumulation in *siz1-2* and *siz1 snc1-11* at 28°C. (**E**) Normalized gene expression of *PR1*, *PR2* and *SNC1* (mean ± SE) in 5-week-old plants (fold change; Col-0 at 22°C = 1). *PR1* and *PR2* expression are still elevated in *siz1 snc1-11* at 22°C. At 28°C *PR1* expression is gone in *siz1 snc1-11*, while *PR2* expression remains up regulated (7-fold up).

Hence, we assessed if other hallmarks of the SNC1^auto-I^ phenotype are also partially rescued when *siz1* is grown at 28°C. We looked at spontaneous cell death, expression of defence-related genes (*PR1*, *PR2*, and *SNC1*), and accumulation of the encoded PR proteins. Both spontaneous cell death and *PR1* expression are known (*i*) to strongly depend on EDS1/PAD4 and SA accumulation, and (*ii*) to be suppressed at 28°C in the aforementioned SNC1^auto-I^ mutants. Spontaneous cell death was fully suppressed when *siz1* was grown at 28°C (**Fig 1F**). At 22°C spontaneous cell death was lost in the double mutants *siz1 pad4*, *siz1 eds1* and *siz1 NahG* (**Fig 1F**), indicating that EDS1/PAD4 and SA accumulation are required for the spontaneous cell death in *siz1*. At 22°C expression of *PR1* and *PR2* was also strongly up-regulated in *siz1* compared to the control (Col-0) and expression of both genes required EDS1, PAD4 and SA accumulation (**Figs 2E, S1A and S1B**). At 28°C, *PR1* expression was completely suppressed in *siz1*, but *PR2* expression partially remained (**S1B Fig**). This situation was reflected in their protein levels, *i*.*e*. PR1 levels were high in *siz1* at 22°C while undetectable at 28°C (**Fig 1G**). In contrast, PR2 levels were elevated in *siz1* both at 22°C and 28°C albeit to a lower level at 28°C. In the case of the four SNC1^auto-I^ mutants, PR1 and PR2 did not accumulate when these mutants were grown at 28°C (**Figs 1G and 2D**). Thus, the *siz1* auto-immune response is (partially) temperature sensitive, but it does not simply mimic the ‘classic’ behaviour of SNC1^auto-I^ mutants.

### Spontaneous cell death but not accumulation of PR2 depends in *siz1* on SNC1

As elevated expression of *SNC1* triggers auto-immunity at 22°C [42], we measured *SNC1* expression in *siz1*. *SNC1* expression proved to be induced by nearly 5-fold in *siz1* at 22°C (**S1C Fig**), suggesting that an increase in *SNC1* transcript levels could be causal for the *siz1* dwarf stature and auto-immunity. To determine if the *SNC1* gene is indeed required for the *siz1* phenotype at 22°C/28°C, we crossed *siz1* with a loss-of-function mutant of *SNC1*, *snc1-11* (SALK_04705). This mutant has a T-DNA insertion in the first exon, which results in a severely truncated transcript [42]. When grown at 22°C, the *siz1 snc1-11* double mutant displayed a small but significant growth recovery compared to *siz1* (**Fig 2A and 2B**; group ‘c’ and ‘d’, respectively), which is more apparent when the plants are flowering (**S2 Fig**). However, in our conditions the *snc1-11* mutant itself also displayed a small but significant increase in biomass compared to the wild type control (Col-0) at 22°C (**Fig 2B**). More importantly, both *siz1* and the *siz1 snc1-11* double mutant largely kept their dwarf stature when grown at 28°C. This is striking, as the growth defects of the SNC1^auto-I^ mutants *cpr1-2*, *bon1* and *srfr1-4* recovered strongly (to wild type levels) when the *snc1-11* mutation was introduced in these mutants by crossing [10,15,18]. The increase in *SNC1* transcript levels can, therefore, not be the main or sole cause of the dwarf stature of *siz1*.

Nonetheless, spontaneous cell death was fully suppressed in *siz1 snc1-11* at 22°C (**Fig 2C**), while PR2 and to a lesser extent PR1 still accumulated in *siz1 snc1-11* at 22°C (**Fig 2D**). Also at 28°C PR2 still accumulated to some extent in *siz1 snc1-11*, similar to *siz1* (**Fig 2D**). The PR1 and PR2 protein levels were again mirrored by their gene expression levels (**Fig 2E**), *i*.*e*. at 22°C the expression of *PR1* was roughly 50% in *siz1 snc1-11* in comparison to *siz1*, which in both cases was fully suppressed when these two mutants were grown at 28°C. On the other hand, *PR2* expression remained detectable when both mutants were grown at 28°C. Also the (truncated) transcript of *SNC1* still accumulated to higher levels in *siz1 snc1-11* than in *siz1*. For *snc1-11*, 2–3 samples showed up-regulation of *PR1* and *PR2*, while the remaining samples 5 samples showed hardly any up-regulation suggesting that the latter samples reflect the general trend.

Increased SNC1 protein levels are known to trigger auto-immunity [11]. SNC1 levels are negatively controlled by the HSP90/SGT1/SRFR1 chaperone-complex of which some components were reported to be SUMO substrates [43,44]. We therefore examined whether *siz1* auto-immunity was attenuated when mutants for SGT1a, SGT1b, and RAR1 were introduced by crossing. Introduction of these mutants in *siz1* (*i*.*e*. *siz1 rar1*, *siz1 sgt1a*^*KO*^ and *siz1 sgt1b*^*eta3*^) partially compromised cell death induction (**S3A Fig**), while it hardly enhanced rosette growth in these *siz1* chaperone double mutants (**S3B Fig**). Hence, the chaperones contribute to the *siz1* phenotype, but they are not essential for spontaneous cell death. Clearly, the *siz1* auto-immune phenotype partially depends on SNC1, but not all of the elements of the auto-immune phenotype disappear when SNC1 is non-functional.

### SIZ1 auto-immunity confers resistance to *Pseudomonas* at high temperature in an EDS1- and SNC1-dependent manner

**Fig 3:**
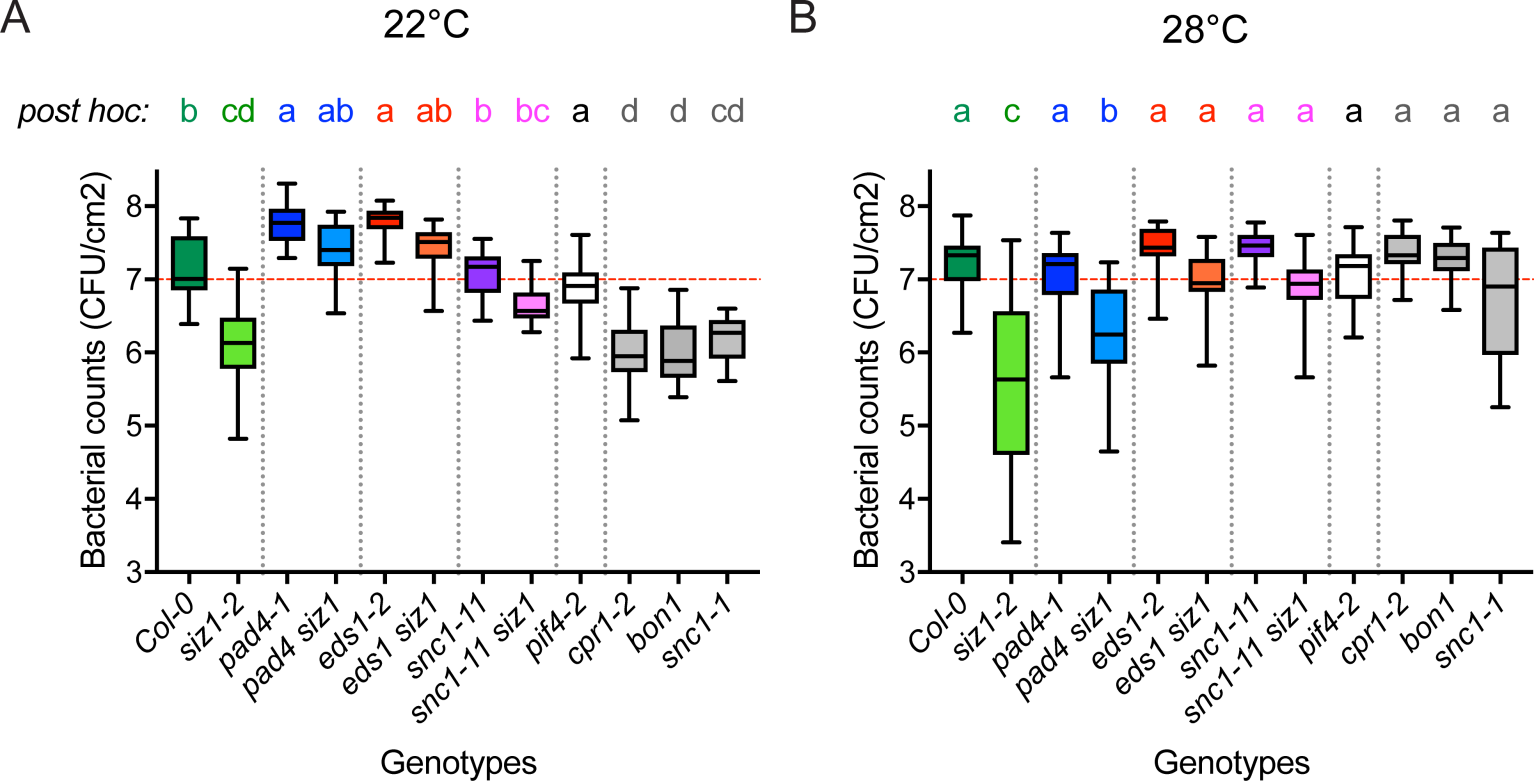
*siz1* displays enhanced resistance to *Pseudomonas* at 22°C and 28°C in a SNC1- and EDS1-dependent manner. **(A)** Disease resistance to *Pseudomonas syringae* pv. *tomato* strain DC3000 (box plot; n = 20–24; Bacterial growth was determined 3 dpi after syringe infiltration of the leaves (1×10^5^ CFU/ml). Significant differences were detected using two-way ANOVA (genotype *p-value* <0.0001, temperature *p-value* = not significant, GxT *p-value* <0.0001) followed by a Tukey post-hoc test. Letters indicate statistically different groups at 22°C. Plants were grown for 5 weeks at 28°C and 24 hrs prior to the inoculation shifted to 22°C. Three independent experiments were combined with each replicate showing the same trend. **(B)** Similar to (A) except that the plants remained at 28°C during the experiment. The letters indicate statistically different groups at 28°C. This experiment was done in parallel with (A).

As the PR1 levels were down in *siz1* at 28°C, we tested if enhanced resistance of *siz1* to the pathogen *Pseudomonas syringae* pv. *syringae* strain DC3000 (PstDC3000) is compromised at high temperature. In order to inoculate similar looking plants, all plants were grown at 28°C and half of the plants was shifted to 22°C twenty-four hours prior to the inoculation. In this way extreme differences in rosette size, morphology, or tissue structure had no impact on the disease assay (compare the plants grown at 28°C in **Fig 1A**). The 24 hours pre-incubation at 22°C was sufficient to re-activate auto-immunity in the SNC1^auto-I^ mutants tested (*cpr1-2*, *bon1*, *snc1-1*) resulting in reduced susceptibility to PstDC3000 (**Fig 3A**, post hoc groups ‘cd’ and ‘d’). As expected, the three tested SNC1^auto-I^ mutants (*cpr1-2*, *bon1*, and *snc1-1*) were as susceptible as the wild type control (Col-0) at 28°C (**Fig 3B**). However, *siz1* displayed enhanced resistance to PstDC3000 both at 22°C and 28°C (**Fig 3A** and **3B**). This resistance was compromised in *siz1* at 22°C when PAD4, EDS1 or SNC1 were mutated (**Fig 3A**). At high temperature, only *siz1 pad4* retained enhanced resistance to PstDC3000, while *siz1 eds1-2* and *siz1 snc1-11* were both as susceptible as the wild type control (**Fig 3B**). This means that enhanced resistance of *siz1* to the pathogen PstDC3000 at 28°C was still dependent on EDS1 and SNC1.

**Fig 4:**
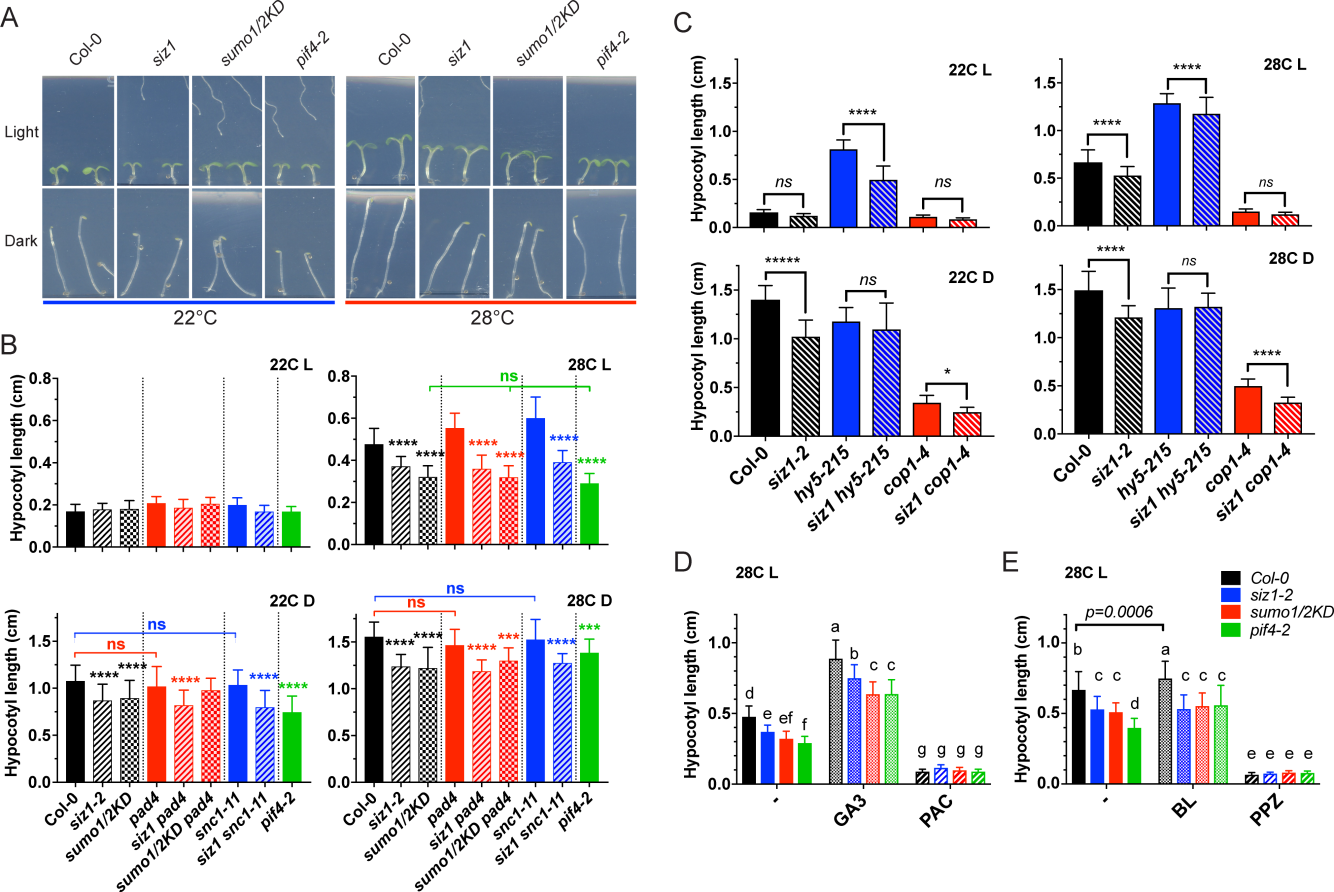
SIZ1 enhances the temperature- and dark-induced hypocotyl elongation independent of PAD4 and SNC1. **(A)** High temperature- and/or dark-induced hypocotyl elongation is compromised in *siz1* and *sumo1/2*^*KD*^. Wild type (Col-0) seedlings and *pif4-2* are shown as positive and negative control for thermosensory hypocotyl elongation, respectively. The pictures were taken 5 d post germination. **(B)** Bar graph (mean ± standard deviation) depicting the hypocotyl length 5 days post germination in 4 conditions: 22C L, 28C L = germination of the seedlings in SD growth conditions at 22°C or 28°C, respectively; 22C D, 28C D = germination of the seedlings in the dark at 22°C or 28°C, respectively. Hypocotyl elongation is significantly reduced in *siz1* and *sumo1/2*^*KD*^ in dark and/or high temperature conditions (compared to Col-0) independent of PAD4 or SNC1. Significant differences were determined using one-way ANOVA (for each condition separately) followed by a Tukey post-hoc test. The brackets indicate the result of the post hoc test for the connected genotypes; otherwise the asterisks denote the difference to the control line (Col-0, *pad4* or *scn1-11*; ****, p≤0.0001; ***, p≤0.001, *, p≤0.05, ns, p>0.05; n = 40–43 samples). The experiment was repeated twice with similar results. **(C)** Bar graph (mean ± standard deviation) depicting the hypocotyl length of *Col-0*, *siz1*, *hy5-215*, *siz1 hy5-215*, *cop1-4*, and *siz1 cop1-4* in response to the same four conditions. SIZ1 is important for hypocotyl elongation of *hy5-215* in a normal dark/light cycles at 22°C (22C L). In dark conditions, loss of SIZ1 enhances the phenotype of the *cop1-4* mutant (22C D, 28C D). Significant differences were determined using one-way ANOVA followed by Tukey post hoc test. The brackets indicate the result of the post hoc test for the connected genotypes. **(D)** Bar graph (mean± standard deviation) depicting the hypocotyl length of the SUMO mutants (*siz1*, *sumo1/2*^*KD*^) in response to GA3 (10 μM) or the GA3 biosynthesis inhibitor PAC (0.5 μM). Gibberellin biosynthesis is needed for hypocotyl growth at 28°C (+PAC), as PAC fully inhibits hypocotyl elongation. Significant differences were determined using two-way ANOVA followed by Tukey post-hoc test; significantly different groups are indicated by the letters (n = 41–44). Seeds were germinated on plate and hypocotyl lengths were measured 5 days post germination at 28°C in SD. *siz1* and *sumo1/2*^*KD*^ showed less germination on PAC (n = 8 and n = 32, respectively). All seeds were fresh and harvested simultaneously. The experiment was repeated twice with similar results **(E)** Similar to (D) except that seeds were germinated on 0.1 μM Brassinolide (BL) or the BR biosynthesis inhibitor PPZ (2.0 μM) with n = 41–52 samples.

In the case of *snc1-1*, high temperature suppression of immunity and restoration of growth were both reported to depend on PIF4 [24]. Therefore, we also tested if the *pif4-2* mutant showed altered resistance to PstDC3000 at 22°C/28°C. The *pif4-2* plants showed a clearly compromised thermomorphogenesis response at 28°C, *i*.*e*. (*i*) the hypocotyl length was reduced (**Fig 4A** and **4B**), (*ii*) the rosette showed no hyponasty and (*iii*) the leaf blades and petioles failed to elongate in comparison to Col-0. However, the *pif4-2* mutant was as susceptible to PstDC3000 as the wild type control (Col-0) at either temperature in our conditions (**Fig 3**).

### Both SIZ1 and SUMO1/2 control dark and high temperature induced hypocotyl elongation

As *snc1-1* auto-immunity is inhibited by PIF4 at high temperature [24], the enhanced immunity of *siz1* to PstDC3000 at 28°C might also be due to reduced PIF4 function. In line with this notion, we found that *siz1* and the *sumo1/2*^*KD*^ mutant both showed reduced hypocotyl elongation at 28°C in normal diurnal dark/light cycles (**Fig 4A**, light; **4B**, compare 22C L with 28C L), implying that SIZ1 and the two archetype SUMO proteins, SUMO1 and SUMO2 (hereafter SUMO1/2), act as positive regulators of thermomorphogenesis similar to PIF4 (*pif4-2* was included as control for the loss of thermosensitive hypocotyl elongation; **Fig 4A** and **4B**). SIZ1 and SUMO1/2 were both also needed for skotomorphogenesis (dark-induced hypocotyl elongation) at 22°C and 28°C (**Fig 4A**, dark; **4B**, compare 22C L with 22C D). The compromised dark and high temperature growth responses were both independent of *PAD4* and *SNC1*, as they still occurred to same extent in *siz1 pad4* and *siz1 snc1-11* (**Fig 4B**). This means that not the auto-immune phenotype of *siz1* is responsible for the compromised thermo/skotomorphogenesis, but rather that SIZ1 itself acts as positive regulator of these growth responses. In support of this notion, we confirmed that the SNC1^auto-I^ mutants *cpr1*, *bon1*, and *srfr1-4* display a normal thermomorphogenesis response (**S4 Fig**), indicating that PIF4 function is unaffected in them. Moreover, the *sumo1/2*^*KD*^ consistently showed a stronger reduction in hypocotyl elongation than *siz1* nearing *pif4-2* at the 28°C in a normal dark/light cycle (**Fig 4B,** 28°C L).

The mutants *siz1* and *sumo1/2*^*KD*^ also displayed a strong reduction in hypocotyl elongation when they were kept in the dark at 22°C and 28°C (**Fig 4B**; panels 22C D, 28C D). As SIZ1 stimulates COP1 activity and the nuclear function of COP1 is activated in the dark [34,35], we examined whether loss of SIZ1 function could enhance the thermo/skotomorphogenesis phenotype of a strong but not lethal COP1 mutant, *cop1-4* [45]. Hypocotyl elongation was indeed more reduced in *siz1 cop1-4* than in *cop1-4* alone in dark conditions at 22°C and 28°C (**Fig 4C**; panels 22C D, 28C D). Thus, COP1 is critical for the thermosensory growth response–as recently reported [27], while SIZ1 appears to primarily enhance this response (as further detailed below).

In light conditions, the TF HY5 is known to inhibit hypocotyl elongation by inhibiting *PIF4* expression [25]. COP1 targets HY5 for proteasomal degradation when COP1 is active in the nucleus. We found that SIZ1 function is needed for the full hypocotyl elongation of the HY5 loss-of-function mutant *hy5-215* in a diurnal light/dark cycle at 22°C (**Fig 4C,** panel 22C L). This means that in a diurnal light/dark cycle at 22°C the stimulatory role of SIZ1 on hypocotyl growth is masked by the inhibitory role of HY5. We also compared the rosette size and morphology of *siz1 cop1-4* and *siz1 hy5-215* with the single mutants at both temperatures (**S5 Fig**). At 22°C *siz1 cop1-4* and *siz1 hy5-215* both adopted a *siz1* rosette size/morphology. At 28°C growth was recovered for *siz1 hy5-215*, but to a lesser extent than for *siz1*. In contrast, *siz1 cop1-4* failed to respond to the high temperature and this mutant still closely resembled *cop1-4* mutant (having a compact rosette with hardly any petioles and no hyponasty; **S5A Fig**). This is consistent with a model in which COP1 primarily conveys the thermosensory growth response and that SIZ1 amplifies the output of this response.

### Hormone biosynthesis is required for the SIZ1-dependent temperature induced growth

As biosynthesis of the hormones gibberellic acid (GA3) and the brassinosteroids is needed for the temperature induced hypocotyl elongation [46], we checked if the positive regulatory role of SIZ1 and SUMO1/2 in thermomorphogenesis requires these two hormones. First, we inhibited GA3 or BR biosynthesis by adding paclobutrazol (PAC) or propiconazole (PPZ), respectively. Irrespective of the genetic background, we found that biosynthesis of both hormones was essential for the temperature-induced hypocotyl elongation in the lines tested including the residual elongation in *pif4-2* (**Fig 4D and 4E**). GA3 is known to reduce the abundance of the DELLAs by triggering their degradation [47]. In turn the DELLAs restrain cell growth by reducing protein abundance of the PIFs (including PIF4) and the TF BZR1 (Brassinazole resistance 1) [48,49]. A combined treatment of 28°C+GA3 resulted in increased hypocotyl elongation for each of the four tested lines compared to the 28°C control (-) (**Fig 4D**). However, hypocotyl elongation was still impaired for *siz1*, *sumo1/2*^*KD*^ and *pif4-2* in the combined treatment 28°C+GA3 (**Fig 4D**). This implies that the positive role of SIZ1 on temperature-induced hypocotyl growth is independent of DELLA accumulation. The combined treatment of 28°C plus the brassinosteroid Brassinolide (28°C+BL) triggered a small but significant increase in hypocotyl elongation in the control (Col-0) plants compared to the mock treatment (**Fig 4E,**—vs. BL). However, the SUMO mutants (*siz1* and *sumo1/2*^*KD*^) showed no additional response to the combined treatment 28°C+BL (**Fig 4E**). Strikingly, the *pif4-2* mutant did respond to the BL treatment (from post hoc group D to C), suggesting that in *siz1* and *sumo1/2*^*KD*^ brassinosteroid signalling is apparently already at its maximum physiological level.

### SIZ1 controls amplitude and timing of the transcriptional response to high temperature in part via PIF4/BZR1

To elucidate how SIZ1 and SUMO1/2 conjugation affect high temperature-induced gene expression, we grew *siz1 pad4* and the *sumo1/2*^*KD*^ *pad4* mutants for two weeks at 22°C and then shifted them to 28°C (4 hrs after light onset) to trigger a temperature induced transcriptional response. To avoid that constitutive (auto-)immune signalling impedes the thermosensory transcriptional response at t = 0, we performed the experiment in the *pad4* background, which largely blocked *siz1* auto-immunity at 22°C (i.e. the enhanced accumulation of PR1 and PR2, spontaneous cell death and the increased resistance to PstDC3000 are suppressed in *siz1 pad4*; **Figs 1F, 1G** and **3A**), but it only partially restored the dwarf stature. Importantly, increased resistance to PstDC3000 was not lost in *siz1 pad4* at 28°C, similar to *siz1* (**Fig 3B**). The plants were sampled at the shift to 28°C (day 0) and 24hrs (day 1) and 96 hrs (day 4) after the shift. Catala *et al*. had previously shown that *siz1* shows a strong up-regulation of defence-related genes (like *PR* genes and immune receptors), while genes involved in BR biosynthesis/signalling are down-regulated [50]. We first determined which genes are differentially expressed at 22°C in *siz1 pad4* in comparison to the control (*pad4*). We found that a small set of genes encoding for TNL immune receptors, Receptor-like kinases (RLKs), and Receptor-like proteins (RLPs) remained up-regulated in *siz1 pad4* in comparison to the control (*pad4*) at 22°C (**S1A Table**). *SNC1* or immune receptors of the CNL type (Coiled-coil NB-LRR-type) were not amongst the up-regulated genes in the microarray data. Real time PCR revealed that *SNC1* was roughly two-fold induced in *siz1 pad4* (close to the cut-off value for differential gene expression), while *SNC1* showed no up regulation in *siz1 eds1* or *siz1 NahG* (**S1C Fig**). As *SNC1* was 5-fold induced in *siz1* at 22°C (**S1C Fig**), we conclude that this requires feedback regulation via EDS1 and SNC1.

There was no broad up-regulation of TF families linked to plant immunity (WKRY, TGA or MYC family) in *siz1 pad4* at 22°C. Likewise, *PR* genes like *PR2*, *PR3*, or *PR4* were no longer strongly up-regulated in *siz1 pad4* at 22°C. The genes involved in BR biosynthesis and signalling were also no longer collectively down-regulated except for two genes, which encode for two rate-limiting enzymes of the Brassinosteroid (BR) biosynthesis pathway (*DWF4* or *DWARF 4;* and *BR6OX2* or *BRASSINOSTEROID-6-OXIDASE 2*) [51–53]. This suggests that the BR levels might be reduced in *siz1 pad4*. In agreement with this, we found that the TFs *BEE1*, *BEE3*, and *TCP1* are down-regulated in *siz1 pad4* (**S1B Table**). BEE1 and -3 are two closely related bHLH TFs that act as early response TFs required for the full BR response [54]. *TCP1* encodes a TF that directly positively regulates the expression of *DWF4* [55]. Combined, these data argue that the *siz1 pad4* phenotype may be (partially) due to BR-deficiency.

**Fig 5:**
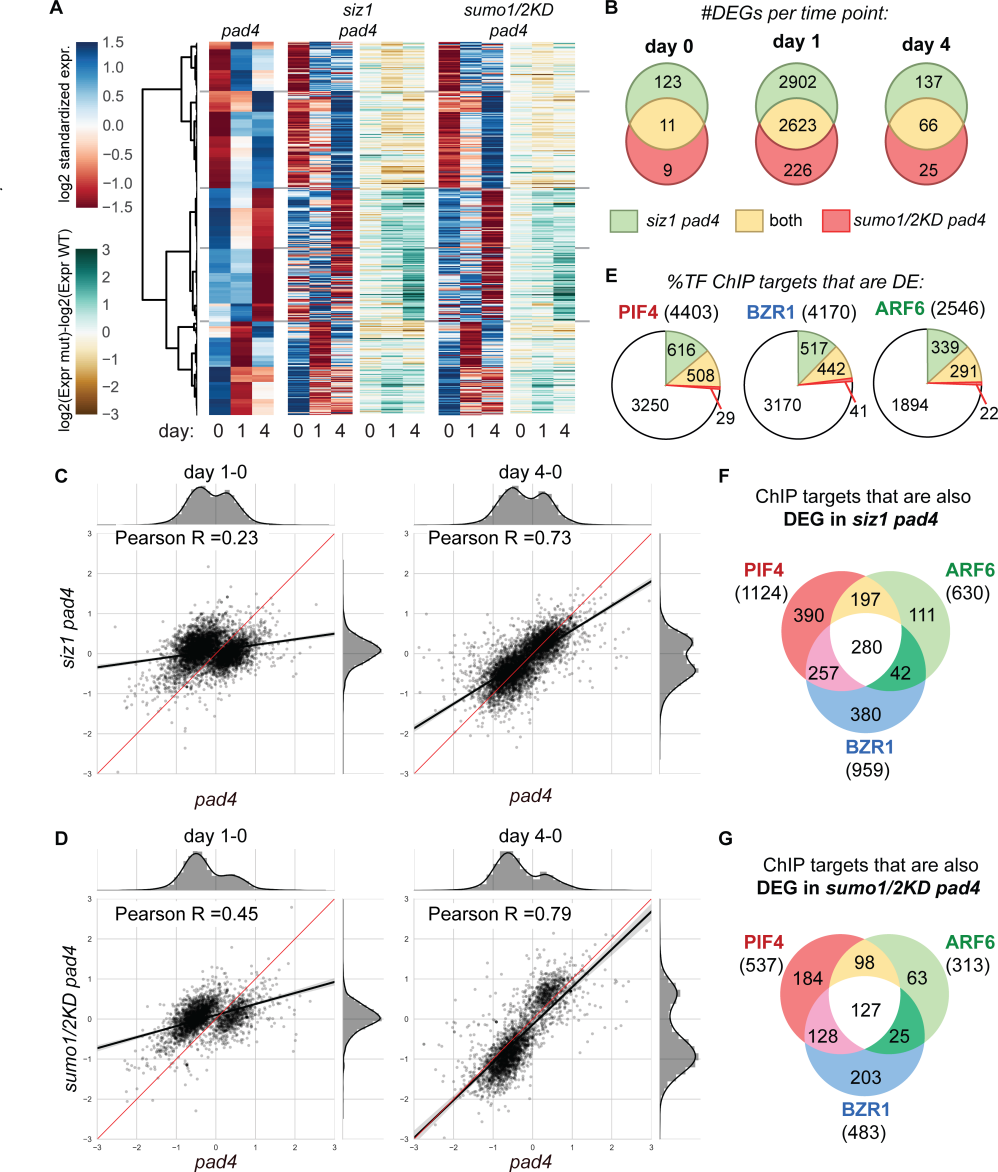
SIZ1 promotes the timing and amplitude of the expression of thermosensitive genes, including genomic targets of the transcription factors PIF4/BZR1. **(A)** Heat maps representing thermosensitive genes, i.e. differentially expressed genes in *pad4* in response to the shift to 28°C and their expression profiles in the *siz1 pad4* and *sumo1/2*^*KD*^ *pad4* mutants (blue-to-red diagrams). Gene clustering was based on Pearson correlation of the expression profiles in *pad4* (shown on the left). To reveal gene expression dynamics over time in the mutants versus *pad4*, the expression values were standardized (zero mean, unit variance per profile). Of note, the expression profiles of the thermosensitive genes were largely similar for the three genotypes. In addition, the relative difference-in-expression (log2 fold change, non-standardized) is shown for the thermosensitive genes in ‘*siz1 pad 4* versus *pad4’* and ‘*sumo1/2*^*KD*^ versus *pad4’* (brown-to-cyan depicting *less expressed-to-more expressed* in the mutant). **(B)** Venn diagrams showing the total number of differentially expressed genes (#DEGs, q≤0.01) in *siz1 pad4* (green), *sumo1/2*^*KD*^ *pad4* (red) and their overlap (yellow) in comparison to *pad4* at the three time points. The largest change in global gene induction/repression occurs at day 1 in both two mutants. **(C)** Scatter plot depicting the change-in-expression at day 1 or 4 with respect to the expression at day 0 (day 1–0; day 4–0) for all the DEGs in *siz1 pad4* (y-axis) in comparison to their change in expression in *pad4* (x-axis). The DEGs are the same as panel (B). For each DEG is shown the log2-fold change-in-expression. The density plots at the right and top depict the global change in expression for these DEGs in the mutant and the wild type, respectively. The black lines depict a Pearson linear regression analysis for all the DEGs with the 95% confidence interval indicated by the grey zone. The red line depicts an equal change in expression for all genes in *siz1 pad4* versus *pad4*. **(D)** Similar to (C), but the scatter plot depicts the change in expression for the DEGs in *sumo1/2*^*KD*^ *pad4* in comparison to *pad4*. **(E)** Pie diagrams depicting the total number of direct genomic targets of PIF4, BZR1 and ARF6, retrieved from published chromatin immuno-precipitation (ChIP) datasets (Adapted from [56]), and the number these targets that are differentially expressed (DE) in *siz1 pad4 (*green+yellow*)* or *sumo1/2*^*KD*^ *pad4* (red+yellow). These two sets are a combination of the DEGs from panel B. The overlap between the genomic targets of these three TFs and the DEGs in *siz1 pad4* and *sumo1/2*^*KD*^ *pad4* was significant (*p-values* for *siz1 pad4*: PIF4, 3.1e-24; BZR1, 2.1e-8; ARF6, 9.2e-11; *p-values* for *sumo1/2*^*KD*^ *pad4*: PIF4, 1.2e-6; BZR1, 6.9e-4; ARF6, 1.5e-4; hypergeometric test). **(F)** Venn diagram depicting the overlap between the genomics targets of PIF4, BZR1 and ARF6 that are differentially expressed in *siz1 pad4* over the course of the experiment. Most DEGs are a target of PIF4 and/or BZR1. **(G)** Venn diagram depicting the overlap between the genomics targets of PIF4, BZR1 and ARF6 that are differentially expressed in *sumo1/2* ^*KD*^ *pad4* over the course of the experiment.

We then selected the set of thermosensitive genes by identifying the genes that are differentially expressed (DEGs, q ≤ 0.01) in *pad4* in response to the shift to 28°C (comparing day 1 to day 0, day 4 to day 1, and day 4 to day 0). The DEGs were clustered based on their expression profile and their expression dynamics was revealed by plotting their standardized expression values in a clustered heat map (**Fig 5A**, *red-to-blue*). To detect differences in the gene expression profiles of *siz1 pad4* and *sumo1/2*^*KD*^ *pad4* we plotted the same gene expression heat maps for the two mutants while retaining the gene clustering (**Fig 5A**). We also plotted the difference in gene expression (Δ) between the mutants (*siz1 pad4* and *sumo1/2*^*KD*^ *pad4*) and *pad4* (*brown-to-cyan* heat maps). **Fig 5A** reveals that overall the gene expression profiles of the thermosensitive genes do not differ strongly between the two SUMO conjugation mutants and the control *pad4* (*blue-to-red* heat maps). In other words, most of the thermosensitive genes also respond to the shift to 28°C in *siz1 pad4* or *sumo1/2*^*KD*^ as they do in *pad4*.

However, most of the thermosensitive genes appear to show an attenuated response in *siz1 pad4* and *sumo1/2*^*KD*^ *pad4* at day 1 and/or 4. For example the up-regulated genes (changing from red at day 0 to blue at day 4 in the heat maps) show less expression in *siz1 pad4* and *sumo1/2*^*KD*^ *pad4* than *pad4* at day 4 (*brown* colour in the ‘ΔExpr (mut-WT)’ heat maps). Likewise, the down-regulated genes (shift from blue at day 0 to red at day 4) show increased expression in *siz1 pad4* and *sumo1/2*^*KD*^ *pad4* at day 4 (*cyan* colour in the ‘ΔExpr (mut-WT)’ heat maps). To confirm this notion, we selected for each time point the DEGs in *siz1 pad4* and *sumo1/2*^*KD*^ *pad4* in comparison to *pad4*. A large set of these DEGs was shared between the two mutants (*siz1 pad4* and *sumo1/2*^*KD*^ *pad4*), as can be seen in the VENN diagrams (**Fig 5B**). Strikingly, the largest number of DEGs was obtained for both mutants at day 1 rather than at day 4. To visualize the dynamic response of these DEGs in response to high temperature, we plotted in a scatter plot the fold change in expression of these DEGs for *siz1 pad4* and *sumo1/2*^*KD*^ *pad4* (both y-axis) versus *pad 4* (x-axis) (by separately combining the DEGs for the different time points for the two mutants). The left panel in **Fig 5C** and **5D** depicts the change in expression from day 0 to day 1, while the right panel depicts the change from day 0 to day 4. This revealed that primarily in the control (*pad4*) at day 1 the expression of the DEGs changed due to the increase in temperature, while in *siz1 pad4* and *sumo1/2*^*KD*^ *pad4* these genes largely failed to respond at this time point (**Fig 5C and 5D**, panel day 1–0). This is best seen in the global expression profiles (top and right side of the scatter plot) revealing a double hump in *pad4*, while the expression profile displays a single Gaussian curve around zero for both SUMO mutants. In contrast, at day 4 we find a positive correlation for the change in expression of all DEGs (Pearson R = 0.73; linear regression) with a slope = 0.61 for *siz1 pad4* versus *pad4*. This means that at day 4 the DEGs responded in *siz1 pad4* to the high temperature, but their response was overall attenuated. A similar situation is seen for *sumo1/2*^*KD*^ *pad4* at day 4 (Pearson R = 0.79; slope = 0.87). Thus, SIZ1 and SUMO1/2 both appear to control in a similar manner both the timing and the amplitude of the temperature-induced transcriptional response.

We then examined if the direct genomic targets of the TFs PIF4/BZR1/ARF6 are differentially expressed in *siz1 pad4* and *sumo1/2*^*KD*^ *pad4*. The direct genomic targets of these tree TFs, which form a trimeric transcriptional hub, were obtained from published chromatin-immunoprecipitation (ChIP) datasets of these TFs [56,57]. As shown in **Fig 5E**, nearly 25% of the genomic targets of these three TFs was differentially expressed in *siz1 pad4* during the course of the temperature shift experiment. This overlap was very significant with *p-values* of 3.07e-21 (PIF4), 2.11e-8 (BZR1), 9.24e-11 (ARF6) using a hypergeometric test (based on 26859 annotated probes; TAIR9). The overlap was still significant but less strong for *sumo1/2*^*KD*^ *pad4* (with an overlap of ±12%, **Fig 5E**) and *p-value*s of 1.17e-6 (PIF4), 6.92e-4 (BZR1), 1.46e-4 (ARF6). Thus, there is a significant enrichment for the genomic targets of these three TFs amongst the DEGs in both our mutants in response to shift to temperature 28°C (**Fig 5E**). The change in expression of these genomic targets of these three TFs in the mutants versus the control (*pad4*) mirrored largely the global pattern seen for all the DEGs combined (**S6** and **S7 Figs**). Thus, the response of the misexpressed genomic targets of PIF4, BZR1, and ARF6 in the *siz1 pad4* and *sumo1/2*^*KD*^ *pad4* mutants follows the same trend as the global response (i.e. their expression is largely delayed till day 4 and the response remains attenuated at day 4). This corroborates our hypothesis that the PIF4-dependent high-temperature growth response is compromised in *siz1* and *sumo1/2*^*KD*^. We also looked at the genomic targets of the ‘cold’ regulator HY5 that binds to and competes (at low temperature) for the same genomic targets as PIF4 [58,59]. The HY5 genomic targets largely failed to respond in *siz1 pad4* at day 1, while at day 4 their response was largely attenuated in *siz1 pad4* compared to *pad4* (**S8C** and **S8D Fig**). This effect on the expression of the HY5 genomic targets was less clear for *sumo1/2*^*KD*^ *pad4* (**S9C** and **S9D Fig**). While examining the list of DEGs we noted that many *SAUR (Small auxin up RNA)* genes were present among the top of the gene lists. PIF4 is known to regulate auxin biosynthesis via the *SAUR* family [60]. The differentially expressed *SAUR* genes showed a strong deregulation in *siz1 pad4* and *sumo1/2*^*KD*^ *pad4* at both time points, with very distinct global expression profiles in the mutants versus the control (*pad4*) (**S8E, S8F, S9E and S9F Figs)**. Combined, our data revealed that the *siz1 pad4* and the *sumo1/2*^*KD*^ *pad4* mutants display a delayed and attenuated transcriptional response to high temperature (in comparison to *pad4*), which runs in part over the PIF4/BZR1 transcriptional hub.

## Discussion

**Fig 6:**
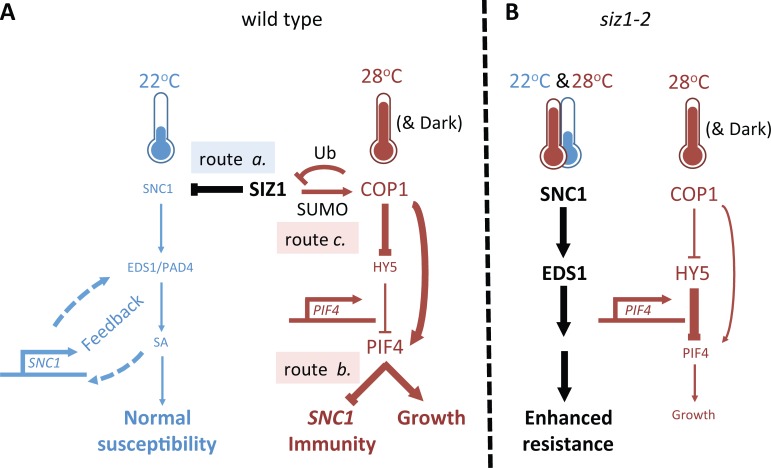
Model depicting the role of SIZ1 in (*i*) SNC1-dependent auto-immunity and (*ii*) thermosensory growth via COP1 and PIF4. **(A)** Wild type situation; SIZ1 inhibits at the transcription and/or protein level SNC1-dependent auto-immunity (route *a*.). This involves PAD4, EDS1, SA accumulation, and transcriptional feedback regulation. SNC1 auto-immunity is suppressed at 28°C by PIF4 function, at least for the mutant *snc1-1* (route *b*.) [24]. SIZ1 sumoylation of COP1 stimulates the intrinsic ubiquitin (Ub) E3 ligase activity of COP1 resulting in degradation of COP1 substrates, including HY5 and SIZ1 [34,35] (route *c*.). In this way, SIZ1 amplifies and tunes COP1 activity at high temperature and/or dark conditions (based on **Fig 4**). **(B)** In the *siz1-2* mutant, auto-immunity is not inhibited at low and only partially at high temperature. This results in enhanced resistance to bacteria in *siz1-2* at low and high temperature, while requiring EDS1 and SNC1 function (**Fig 3**). COP1 activity is required to convey thermosensing resulting in less hypocotyl elongation growth in *cop1-4* mutant (Fig 4C). This (residual) COP1 activity is reduced when SIZ1 is mutated (based on **Fig 4C**; [34,35]). As inhibition of *snc1-1* auto-immunity at high temperature requires PIF4 function, it appears that route b. is compromised or absent in the *siz1-2* mutant. The blue/red/black arrows depict signalling routes at 22°C, 28°C, or that are independent of these temperatures, respectively. The thickness of the arrows marks the amount of protein activity.

Here, we describe an interconnected dual role for SIZ1 and SUMO1/2 conjugation in the switch between plant immunity and high temperature induced growth (as summarized in the model of **Fig 6**). Our data unveil that both SIZ1 and SUMO1/2 conjugation are positive regulators of thermo- and skotomorphogenesis upstream of the PIF4/BZR1 growth regulation hub. In this hub, BZR1 is activated by the hormone BL, while PIF4 is activated by dark conditions and high ambient temperature. In line, these two TFs share a large number of genomic targets that are synergistically regulated by them [56]. We find that loss of SIZ1 and SUMO1/2 both delays and attenuates this transcriptional response to high temperature affecting many targets of PIF4 and BZR1. This suggests that SIZ1 activity acts as a positive regulator of PIF4 function in thermomorphogenesis and that PIF4 function is apparently compromised/inhibited in *siz1* at high temperature (**Fig 6**, *siz1-2*). Importantly, the PIF4 protein abundance is positively regulated by COP1 E3 ligase activity [58], while COP1 activity is stimulated by SIZ1-dependent sumoylation (**Fig 6**, wild type route c.) [34,35]. Our data unveil that COP1 is essential to convey this high temperature signal, as recently reported by others [27], while SIZ1 enhances the high temperature and dark signal. This role of SIZ1 in thermo/skotomorphogenesis is distinct from its reported role on cell elongation due to constitutive defence signalling [61], as hypocotyl elongation was still compromised at high temperature when PAD4 or SNC1 were mutated. Likewise, we noted that the rosette of *siz1 pad4*, *siz1 eds1*, and *siz1 NahG* remained compact at 28°C (without strong petiole elongation or hyponasty as seen for Col-0).

Interestingly, part of the *siz1* auto-immune phenotype is sustained at high temperature resulting in enhanced resistance to bacteria (**Fig 3**). This enhanced resistance still required SNC1 and EDS1 function at 28°C (**Fig 3, Fig 6** wild type route a.). The latter is relevant, as both SNC1 and EDS1 immune signalling depend on their nuclear localization, while SNC1 nuclear localization is impaired at high temperature [7,12,22,62]. High temperature suppression of *snc1-1* auto-immunity and concomitantly rescue of its growth phenotype requires PIF4 function [24] (**Fig 6** wild type route b.). SNC1-dependent auto-immunity, including enhanced resistance to the bacterial pathogen *Pseudomonas*, is normally fully suppressed in the mutants *bon1*, *crp1-2* and *snc1-1* at 28°C, resulting in normal rosette growth (e.g. **Figs 1–3**) [7,63]. However, *siz1* fails to resume normal growth at 28°C and this is independent of PAD4/EDS1, SNC1 or SA accumulation. This implies that the ‘high temperature’ signal is not properly conveyed in *siz1*. At the same time, SIZ1 suppresses expression of a small subset of immune receptors at 22°C, even when PAD4 is mutated. It remains an open question if elevated expression of one of these immune receptors (**S1A Table**) is causal for the auto-immune phenotype of *siz1*, rather than the misexpression of SNC1.

Biochemically, SUMO conjugation was already implied as a regulator of photomorphogenesis [34,35]. Our data suggest that the role of SIZ1 in thermomorphogenesis is mechanistically independent of light sensing, as hypocotyl elongation in *siz1* was also reduced in the dark. Previous works had indicated that sumoylation of phyB allows PIF5 to bind its target promoters resulting in root growth stimulation. These authors demonstrated that sumoylation of the Pfr state (red light activated state) of phyB suppresses the interaction between phyB and PIF5, the closest homologue of PIF4 [32,64,65]. Our GA3 treatment experiment also suggests that SIZ1 controls thermomorphogenesis response independent of DELLA accumulation (**Fig 4D**). The DELLAs control the stability of the PIFs, while they themselves are also controlled by sumoylation [31,49]. Other (putative) sumoylation substrates implicated in PIF4 function are ELF3 (Early flowering 3) [43,66], HFR1 [67] and LAF1 [68], HY5 and HY5-like (HYL) [69]. The role of sumoylation has not yet been determined for ELF3. Both HFR1 and LAF1 are also targets for COP1-mediated degradation. The link between their degradation and sumoylation remains to be studied. Nevertheless, it is evident that (i) SUMO conjugation acts at multiple levels as a regulator of growth and that (ii) certain COP1 substrates are also targets for sumoylation.

Finally, we found that several actors in BR biosynthesis and signalling are still down-regulated (*DWF4*, *BEE1*, *BEE3*, and *TCP1*) in *siz1 pad4*. CESTA, a close homologue of BEE1 and BEE3, is another SUMO substrate that directly binds to BEE1 to control BR biosynthesis [70]. Catala and co-workers had previously reported that from the nearly 1600 differentially expressed genes in *siz1* (>two-fold change), eleven down-regulated genes were known to be critical for BR biosynthesis and signalling [50]. In addition, they found in their genome-wide expression analysis that both *PIF4* and *PIF5* were underexpressed in *siz1* [50]. These data warrant further research on the role of sumoylation on BL signalling and biosynthesis.

To conclude, SIZ1 and SUMO1/2 both act as important positive regulators of growth, while SIZ1 also acts as negative regulator of an SNC1-dependent immune response at high temperature. SIZ1 thus plays an interdependent dual role in growth and immunity at elevated ambient temperature.

## Materials and methods

### Plant materials and growth conditions

The genetic resources for this research were wild type Arabidopsis (*Arabidopsis thaliana*) ecotype Col-0, *siz1-2* [71], *cop1-4* [45], *cpr1-2* [18], *bon1-1* [10], *hy5-215* [72], *snc1-1* [19], *srfr1-4* [15], *pad4-1* [73], *eds1-2* (backcrossed in Col-0) [74], *sid2-1* [40], *35Spro*::*NahG* [75], *snc1-11* (SALK_047058) [10], *sgt1a-3* [16], *sgt1b(eta3)* [76], *rar1-21* [77], *pif4-2* [78], and *sumo1/2*^*KD*^ [aka *sum1-1 amiR-SUMO2* line B][29,79]. The double mutants *pad4 siz1, NahG siz1 [28], cop1-4 siz1-2* and *hy5-215 siz1-2* [35] are described elsewhere. Arabidopsis plants were grown under white light with 120 μmol m^-2^ sec^-1^ under short-day (SD) light conditions (11 hr light, 13 hr dark) at 22°C or 28°C on a compost/perlite soil mixture. After crossing, the plants were genotyped according to the primer combinations and primer sequences presented in the **S2 and S3 Tables**, respectively.

### Fresh rosette weight measurements

The fresh rosette weight of plants (minimum 8) grown individually in single pots was measured. The rosette was sampled from 5-week-old plants grown in parallel at 22°C or 28°C. Statistical analyses were made using two-way ANOVA (genotype, temperature, interaction GxT) followed by Tukey post hoc test in Prism7. Significantly different groups are indicated by letters.

### Protein analysis

For immunoblot analysis, seedlings or leaf material was homogenized in liquid nitrogen, thawed on ice in extraction buffer (10% glycerol, 50 mM K_2_HPO_4_/KH_2_PO_4_ pH 7.5, 150 mM NaCl, 1 mM EDTA, 2% w/v polyvinylpolypyrrolidone K25, 1× protease inhibitors (Roche), 1% v/v Nonidet P-40, 0.1% SDS and 5 mM DTT), and centrifuged for 10 min at 13,000*g*. The supernatant was mixed 1:1 with 2× SB (125 mM Tris-HCl pH 6.8, 4% SDS, 20% v/v glycerol, and 100 mM DTT), and the samples were boiled for 10 min. Proteins were separated on 15% SDS-PAGE and blotted onto Polyvinylidene fluoride (Immobilon-P, MIllipore) membranes. Secondary immunoglobulins conjugated to horseradish peroxidase were visualized using ECL Plus (GE Healthcare). Primary antibodies against PR1 (αPR1) were described previously [80] and αPR2 was obtained from Agrisera (#AS12 2366, ~35kDA). Incubation of both primary and secondary antibodies were done in Tris-buffered saline with 0.05% Tween-20 (TBST) followed by three rinses of 10 minutes in TBS. Equal protein loading was confirmed for the samples by Ponceau S staining of the membranes and when needed the loaded total protein amounts were standardized using BCA protein analysis on the total protein extracts prior to protein loading of the gels. The primary antibodies αPR1, αPR2 and the secondary antibody Goat-anti-Rabbit HRP (Fisher) were used at 1:5000, 1:2000 and 1:5000 dilutions, respectively.

### Quantitative gene expression analysis

For the gene expression analysis, total RNA was extracted from 100–200 mg of leaf material of 5-week-old plants grown at 22/28°C using TRIzol LS reagent (Fisher). The RNA was treated with DNase (ThermoFisher) according to the supplier’s protocol and RNA concentrations were determined by measuring the Abs(260) on a Nanodrop. cDNA was synthesised from 1 μg total RNA using RevertAid H reverse transcriptase in the presence of the RNAse inhibitor Ribolock (both ThermoFisher) following the supplier’s protocol. All biological samples were measured in technical replicate with 3–4 biological replicates per experiment. The PCR amplification was followed using Hot FIREPol EvaGreen qPCR (Solis Biodyne) in a QuantoStudio3 (ThermoFisher). Gene expression was normalized using two genes: *Actin2* (At3g18780) and *beta-Tub4* (At5g44340). The primers used are given in the **S2 Table.** The Ct values were corrected for primer efficiencies. All expression data were analysed using the pipeline in qBASE+ (Biogazelle).

### Hypocotyl elongation measurements

Cold-stratified (3 days at 4°C) sterilized seeds (~50 per line) were placed on vertical plates with 1/2 MS medium supplemented with 1% w/v sucrose and 1% w/v Daishin agar (Duchefa). Seeds were irradiated with white light for 6 hrs to promote germination and then incubated in the specified light/temperature conditions for 5 days. The used seeds were fresh and from the same seed harvest. Seedlings were scanned and the hypocotyl lengths were measured using ImageJ (http://rsb.info.nih.gove/ij). Sensitivity to the Gibberellin biosynthesis inhibitor Paclobutrazol (Pac, Duchefa) and the hormone Gibberellic acid (GA3, Duchefa) was analysed by growing the seedlings on 0.5 μM PAC or 10 μM GA3, respectively. Likewise, sensitivity to the Brassinosteroid biosynthesis inhibitor Propiconazole (PPZ, Sigma-Aldrich) or the hormone 24-epiBrassinolide (BL, #b1439, Sigma-aldrich) was analysed by adding 2 μM PPZ or 0.1 μM BL to the plates, respectively.

### Pseudomonas disease assay

*Pseudomonas syringae* pv. *tomato* DC3000 (PstDC3000) [81] (carrying the empty vector pVSP61) was freshly grown overnight at 28°C with 200 rpm in 10 mL Kings B broth [82] supplemented with rifampicin (50 μg/mL) and kanamycin (40 μg/mL) to reach an OD600 of ~0.9–1.2. Directly prior to infiltration, the bacterial suspensions were spun down, washed with 10 mM MgSO_4_, and resuspended at OD600 = 0.0002 (1×10^5^ CFU/mL) in 10 mM MgSO_4_ for syringe leaf infiltrations. For the Pst disease assays the plants were germinated and grown at 28°C constant temperature (with 11L/13D) for 5 weeks in soil. Twenty-four hours prior to inoculation (9:00 am), one batch of plants was moved to 22°C (SD) and both plant sets were placed in propagators to increase humidity (>90%). The two plant batches were simultaneously infiltrated at 22°C and 28°C using the same bacterial suspension. Upon infiltration the plants were left to dry for 1.5–2 hrs after which they were again covered with lids for 72 hours to increase humidity (>90% relative humidity). Humidity and temperature was followed using a data logger inside the propagators for the duration of the experiment. Leaf discs were taken 1 hour after dipping (t = 0) at both temperatures and 72 hrs post infiltration (t = 3). At least 6 plants were infiltrated per condition. In total 8 samples were taken for each condition combining 2–3 leaf discs with a diameter of 5 mm. Leaf discs were taken from different leaves and only ‘mature’ fully elongated rosette leaves were sampled. The first-formed round shaped leaves were excluded from tissue sampling. The sampled intact leaves were surface-sterilized prior to taking leaf discs (10 sec dip in 70% ethanol followed by two washes with sterile water). The disease assays were performed with at least two independent replicates with similar results.

### Cell death analyses using trypan blue staining

The rosette leaves were stained with a 1:1 mixture (v/v) of ethanol and lactic acid–phenol–trypan blue solution (2.5 mg mL^−1^ trypan blue, 25% v/v lactic acid, 25% phenol, 25% glycerol, and water) and boiled for 5 min. For destaining, the trypan blue solution was replaced with a chloral hydrate solution (2.5 g mL^−1^ in water), as described [83].

### Microarray gene expression analysis

The *siz1 pad4*, *sumo1/2*^*KD*^ *pad4*, and *pad4* plants were grown on soil in SD conditions at 22°C for 2 weeks and then transferred to 28°C at noon (t = 0). Leaf samples were taken in triplicate for total RNA extraction at t = 0, 24 hrs (1d), and 96 hrs (4d). Total RNA was purified using the RNAeasy mini kit (QIAGEN). The RNA quality was examined by monitoring Abs(260/280) and the Abs(260/230) ratios. Total RNA (100 ng) was amplified using the GeneChip WT PLUS kit (Affymetrix) generating biotinylated sense-strand DNA targets. The labelled samples were hybridized to Arabidopsis Gene 1.1 ST arrays (Affymetrix). Washing, staining and scanning was performed using the GeneTitan Hybridization, wash, and stain kit for WT Array Plates, and the GeneTitan Instrument (both Affymetrix).

All arrays were subjected to a set of quality control checks, such as visual inspection of the scans, checking for spatial effects through pseudo-color plots, and inspection of pre- and post-normalized data with box plots, ratio-intensity plots and principal component analysis. Normalized expression values were calculated using the robust multi-array average (RMA) algorithm [84]. The experimental groups were contrasted to test for differential gene expression. Empirical Bayes test statistics were used for hypothesis testing [85] using the Limma package in R 3.2.1 (http://cran.r-project.org/), and all *p-values* were corrected for false discoveries according to Storey and Tibshirani [86]. Downstream statistical analyses (*e*.*g*. hypergeometric tests on enrichment) were performed in Python using the Scipy.stats module (https://scipy.org/scipylib/). The microarray data were deposited in Gene Expression Omnibus (GEO, http://www.ncbi.nlm.nih.gov/geo/) under the accession number GSE97641 and Github (DEGs and scripts used to prepare **Fig 5**; https://github.com/LikeFokkens/Siz1_immunity-vs-growth_temperature).

## Supporting information

S1 Table SIZ1 inhibits expression of several immune receptors independent of PAD4, while allowing expression of two rate-limiting enzymes in Brassinosteroid synthesis. **(A)** List of genes encoding TNLs, Receptor-like kinases (RLKs), and Receptor-like proteins (RLPs) genes whose expression is induced in *siz1 pad4* compared t*o pad4* at 22°C, ranked by fold change. Up-regulation of these genes is SIZ1-dependent while independent of PAD4. Statistical significant differences are indicated with *q values*.
**(B)** Similar to (A). Brassinosteroid biosynthesis is possibly reduced in *siz1 pad4*. Expression of the genes *DWF4*, *BR6OX2*, *BEE1*, *BEE3*, and *TCP1* is down-regulated in *siz1 pad4* relative to *pad4* at 22°C. DWF4 and BR6OX2 catalyse two rate-limiting reactions of brassinosteroid biosynthesis. BEE1, BEE3 and TCP1 are TFs involved in brassinosteroid signalling. Statistical significances differences are indicated with *q values*.
(DOC)

S2 Table Primers used in this study. (DOC)

S3 Table Primer combinations used to genotype the different Arabidopsis alleles used in this study. (DOC)

S1 Fig EDS1, PAD4 and SA accumulation are needed for full induction of marker genes of defence and *SNC1* in the mutant *siz1*. Normalized gene expression of the defence marker genes *PR1* (A), *PR2* (B) and *SNC1* (C) using qRT-PCR (mean ± SE, Col-0 at 22°C = 1). RNA was isolated from 5-week-old plants. 3–4 biological replicates were measured in technical replicate. The experiment was repeated twice and the data combined. Experiment is part of the same set shown in **Fig 2E**.
(JPG)

S2 Fig The *siz1* dwarf phenotype partially depends on SNC1 at 22°C. The picture was taken using 6-week-old flowering plants.
(JPG)

S3 Fig The chaperones SGT1a, SGT1b and RAR1 are partially required for the auto-immune phenotype of *siz1*. **(A)** Loss-of-function mutants of *RAR1*, *SGT1a* and *SGT1b* were introduced in *siz1-2* by crossing. The double mutants show less cell death than the *siz1* single mutant. *siz1 snc1-11* is included as neg. control (see **Fig 2C**). Leaves of 5-week-old plants were stained with Trypan blue. To quantify cell death the number of lesions was counted per leaf size area for each genotype. At least 10 images were counted per genotype. Statistical analyses were made using an unpaired two-sided student t-test (grey lines) with ns for p>0.05; * for p≤0.05; ** for p≤0.01 and *** for p≤0.001.
**(B)** Introduction of loss-of-function mutants of *RAR1*, *SGT1a* and *SGT1b* in *siz1* hardly rescues the growth retardation of *siz1*. Rosette weight was taken from 5-week-old plants (n = 8).
(JPG)

S4 Fig Hypocotyl elongation as part of the skoto- and thermomorphogenesis response is not compromised as a result of SNC1-dependent auto-immunity. Whereas hypocotyl growth is compromised in *siz1* and *sumo1/2*^*KD*^, the mutants *cpr1*, *bon1*, and *srfr1-4* show normal hypocotyl elongation at elevated temperature (both in a diurnal cycle and in dark conditions; 28C L and 28C D, respectively). Only *srfr1-4* shows less hypocotyl elongation in dark conditions at 22°C (28C D). Seeds were germinated on plates at 22°C/28°C in SD (L) or dark (D) conditions. Hypocotyl length was measured 5 days post germination. Significant differences were determined using ANOVA followed by Tukey post-hoc test (****, p≤0.0001; ***, p≤0.001; ns, p>0.05; n = 40–43). All significant differences indicated are in comparison to Col-0 (control). The result shown was part of the experiment in **Fig 4A** and **4B**. Experiment was repeated two times with similar results. Error bars indicate standard deviation.
(JPG)

S5 Fig Growth retardation of *siz1* is partially rescued at 28°C by loss of HY5 function, while COP1 is needed for growth at both 22°C and 28°C. **(A)** Picture of the rosettes of *siz1 hy5-215*, *siz1 cop1-4* and the single mutants. Plants were grown for 5 weeks at 22°C or 28°C (SD). The double mutants adopted a similar morphology as *siz1-2* at 22°C. while the growth retardation of *siz1 hy5-215* partially recovered at 28°C albeit slightly less than *siz1* alone. The rosette of *siz1 cop1-4* remained as compact as *cop1-4* alone without petiole elongation at 28°C, indicative of a compromised thermomorphogenesis response.
**(B)** Box-plot (middle bar = median, box limit = upper and lower quartile, extremes = Min and Max values) showing the rosette weight of the genotypes depicted in (A). Weight was taken from 5-week-old plants. Significant differences were detected using a two-way ANOVA with Tukey’s multiple comparisons test; the letters indicate significantly different groups (n = 8–10). The experiment was repeated twice times with similar result.
(JPG)

S6 Fig The expression profile of the genomic targets of PIF4, BZR1, and ARF6 is altered in *siz1 pad4* in response to high temperature. **(A, B)** Scatter plot showing the log2 fold change in expression of all DEGs (black spots) at the three time points in *siz1 pad4* versus *pad4* (identical to **Fig 5C** and **5D**) for [day 1–0] and [day 4–0], respectively. The black line depicts a Pearson linear regression result on the DEGs with the 95% confidence interval indicated by the grey zone.
**(C, D)** Similar to (A, B) except that only the DEGs are shown that are also genomic targets for binding of PIF4 (red spots), BZR1 (blue spots) or ARF6 (green spots), *top-to-bottom*. The red, blue and green lines depict the Pearson linear regression analysis on these DEGs that are also genomic targets of these different TFs with the 95% confidence interval indicated by the red, blue or green zone.
(JPG)

S7 Fig The expression profile of the genomic targets of PIF4, BZR1 and ARF6 is altered in *sumo1/2KD pad4* in response to high temperature. **(A, B)** Scatter plot showing the log2 fold change in expression of all DEGs (black spots) at the three time points in *sumo1/2*^*KD*^ *pad4* versus *pad4* (identical to **Fig 5C** and **5D**) for [day 1–0] and [day 4–0], respectively. The black lines depict a linear Pearson regression analysis on the DEGs with the 95% confidence interval indicated by the grey zone.
**(C, D)** Similar to (A, B) except that only the DEGs are shown that are also genomic targets for binding of PIF4 (red spots), BZR1 (blue spots) or ARF6 (green spots), *top-to-bottom*. The red, blue and green lines depict a Pearson linear regression analysis on these DEGs that are also genomic targets of PIF4, BZR1 or ARF6, respectively, with the 95% confidence interval indicated by the red, blue or green zone.
(JPG)

S8 Fig The expression profile of genomic targets of HY5 and the *SAUR* genes is altered in *siz1 pad4* in response to high temperature. **(A, B)** Scatter plot showing the log2 fold change in expression of all DEGs (black spots) at the three time points in *siz1 pad4* versus *pad4* (identical to **Fig 5C** and **5D**) for [day 1–0] and [day 4–0], respectively. The black lines depict a Pearson linear regression analysis of the differentially expressed genes with the 95% confidence interval indicated by the grey zone.
**(C, D)** Similar to (A, B) except that only the DEGs are shown that are also a genomic target for binding of HY5 (purple spots). The purple line depicts a Pearson linear regression analysis on these DEGs that are also genomic targets of HY5, with the 95% confidence interval indicated by the purple zone.
**(E, F)** Similar to (A, B) except that only the DEGs are shown that encode for *SAUR* genes (yellow spots). These *SAUR* genes are clearly up-regulated in *pad4* at day 1 and 4, but they fail to respond at day 1 and their response is irregular at 4 day in *siz pad4*. The yellow line depicts a Pearson linear regression analysis on these DEGs that encode *SAURs* with the 95% confidence interval indicated by the yellow zone.
(JPG)

S9 Fig The expression profile of genomic targets of HY5 and *SAUR* genes is altered in *sumo1/2KD pad4* in response to high temperature. **(A, B)** Scatter plot showing the log2 fold change in expression of all DEGs (black spots) at the three time points in *sumo1/2*^*KD*^ *pad4* versus *pad4* (identical to **Fig 5C** and **5D**) for [day 1–0] and [day 4–0], respectively. The black lines depict a Pearson linear regression analysis on the differentially expressed genes with the 95% confidence interval indicated by the grey zone.
**(C, D)** Similar to (A, B) except that only the DEGs are shown that are also a genomic target for binding of HY5 (purple spots). The purple line depicts a Pearson linear regression analysis on the DEGs that are also genomic targets of HY5, with the 95% confidence interval indicated by the purple zone.
**(E, F)** Similar to (A, B) except that only the DEGs are shown that encode for *SAUR* genes (yellow spots). The yellow line depicts a Pearson linear regression analysis on these DEGs that encode *SAURs* with the 95% confidence interval indicated by the yellow zone.
(JPG)
