# Supplementary material for: The Arabidopsis SUMO E3 ligase SIZ1 mediates the temperature dependent trade-off between plant immunity and growth
Source: PLoS Genet. 2018 Jan 22;14(1):e1007157. doi: 10.1371/journal.pgen.1007157 (PMC5794169; doi:10.1371/journal.pgen.1007157)
Supplement: S1 Table — (A) List of genes encoding TNLs, Receptor-like kinases (RLKs), and Receptor-like proteins (RLPs) genes whose expression is induced in siz1 pad4 compared to pad4 at 22°C, ranked by fold change. Up-regulation of these genes is SIZ1-dependent while independent of PAD4. Statistical significant differences are indicated with q values. (B) Similar to (A). Brassinosteroid biosynthesis is possibly reduced in siz1 pad4. Expression of the genes DWF4, BR6OX2, BEE1, BEE3, and TCP1 is down-regulated in siz1 pad4 relative to pad4 at 22°C. DWF4 and BR6OX2 catalyse two rate-limiting reactions of brassinosteroid biosynthesis. BEE1, BEE3 and TCP1 are TFs involved in brassinosteroid signalling. Statistical significances differences are indicated with q values. (DOC) [file pgen.1007157.s001.doc]

**S1 Supplemenatary table.** Up-regulation of genes encoding (putative) immune receptors and down-regulation of genes implicated in the Brassinosteroid pathway that potentially contribute to *siz1* phenotype at normal ambient temperature.

Table S1A. List of TNL-type, Receptor-Like Kinases (RLK), Leucine-Rich Repeats (LRR) and Receptor-Like Proteins (RLP) genes up-regulated in *siz1 pad4* with respect to *pad4* at 22°C.

| **Identifier** | **Protein class** | **Name** | **Fold change** | **q value** |
| --- | --- | --- | --- | --- |
| AT5G46500 | TNL |  | 5.01 | 0.000174075 |
| AT2G32680 | RLP | RLP23 | 4.37 | 0.002882953 |
| AT3G25010 | RLP | RLP41 | 4.26 | 0.008026572 |
| AT4G18250 | RLK | RLK Thaumatin | 4.02 | 0.001019706 |
| AT1G35710 | LRR-RLK | LRR-RLK | 3.83 | 0.000314484 |
| AT1G71400 | RLP | RLP12 | 3.67 | 0.009569854 |
| AT5G46490 | TNL |  | 3.58 | 0.000037600 |
| AT5G38850 | TNL |  | 2.89 | 0.001639992 |
| AT1G29720 | LRR-RLK |  | 2.83 | 0.00010035 |
| AT1G33600 | RLP |  | 2.43 | 0.001931635 |
| AT1G56140 | LRR RLK |  | 2.35 | 0.001569676 |
| AT4G39270 | LRR RLK |  | 2.29 | 0.003351148 |
| AT2G34930 | RLP |  | 2.25 | 0.002075849 |
| AT5G41750 | TNL |  | 2.24 | 0.024082064 |
| AT5G39020 | RLK |  | 2.21 | 0.002982317 |
| AT1G56120 | LRR RLK |  | 2.18 | 0.004604087 |
| AT5G10520 | RLK | RBK1 | 2.17 | 0.015341279 |
| AT4G11460 | RLK | CRK30 | 2.15 | 0.018988502 |
| AT1G61360 | RLK |  | 2.09 | 0.015160307 |
| AT4G19530 | TNL |  | 2.02 | 0.030418372 |
| AT1G11350 | RLK | SD1-13 | 2.02 | 0.007600877 |
| AT3G04210 | TNL |  | 2.00 | 0.015662577 |

Table S1B. List of down-regulated genes in *siz1 pad4* with respect to *pad4* at 22°C, which are linked to BR biosynthesis and/or BR signalling.

| **Identifier** | **Protein class** | **Name** | **Fold change** | **q value** |
| --- | --- | --- | --- | --- |
| AT3G50660 | Brassinosteroid synthesis | DWF4 | 0.53 | 0.014284543 |
| AT3G30180 | Brassinosteroid synthesis | BR6OX2 | 0.62 | 0.048858173 |
| AT1G73830 | Brassinosteroid signaling | BEE3 | 0.46 | 0.048858173 |
| AT1G18400 | Brassinosteroid signaling | BEE1 | 0.30 | 0.004604087 |
| AT1G67260 | Brassinosteroid signaling | TCP1 | 0.68 | 0.007024775 |
