# Supplementary material for: The Arabidopsis SUMO E3 ligase SIZ1 mediates the temperature dependent trade-off between plant immunity and growth
Source: PLoS Genet. 2018 Jan 22;14(1):e1007157. doi: 10.1371/journal.pgen.1007157 (PMC5794169; doi:10.1371/journal.pgen.1007157)
Supplement: S2 Table — (DOC) [file pgen.1007157.s002.doc]

**Supplementary table 2.**

| *Primers used for genotyping* | |
| --- | --- |
| **Primer name** | **Nucleotide sequence (5’->3’)** |
| **3363** | TGTAGTATCCATAGAACCTTCCCTCGTAG |
| **3364** | CCAATCTGGCGAGGGTGGAAATAATGAAGCCGCCGCTGCGAGTGATTCTGATGACGAGAATGATTTAG |
| **3278** | ATTTTGCCGATTTCGGAACCACCATC |
| **3366** | CCGCCAGTCTTTATATCTCTGTTT |
| **3367** | AGTCGCATAAGACTAGCTAAGTACT |
| **3368** | TCGCGTATCTGCTTCTCACACA |
| **3369** | TTCGGGCTCCTATTCTGAGGTC |
| **4138** | CCCTTTCTAGTTTCCTTGAGCTAAG |
| **4139** | TCAGGTATCTGTTATTTCATCCATC |
| **3275** | CGTAAGTTCAATCTTGATGCTCTGCAGC |
| **3276** | AGCCTTGCTTCTTCTGCTGGAAGC |
| **3603** | GGCAATCAGCTGTTGCCCGTCTCACTGGTG |
| **3604** | ATATGGAGATAGCTTCTTCTTCTG |
| **3605** | AAGATCATATCCGGCCATAACTG |
| **3788** | AAGAGACGGACCTGCTTCTTC |
| **3789** | CTTCCATTGTTCATGGTTTGG |
| **3370** | TGGTTCACGTAGTGGGCCATCG |
| **3782** | CGACTAATGAGATTTTCCTCCCAGGTGGTTTTT |
| **3783** | CGCAAGAATGGAGAGAGACA |
| **3786** | TTCATCTTAACATTTGGCCTTG |
| **3787** | TTGGCCAGAACTGGTTTCTC |
| *Primers used for real time quantitative PCR* | |
| **Actin2 _F** (At3g18780), 4551 | CTTGCACCAAGCAGCATGAA |
| **Actin2_R** (At3g18780), 4555 | CCGATCCAGACACTGTACTTCCTT |
| **Beta Tub4_F** (At5g44340), 3320 | CAAGATGCTACAGCCGGAGAGGA |
| **Beta Tub4_R** (At5g44340), 3321 | AGAAGAAAGTGAATGCTGCTTGCT |
| **PR1_F** , 3207 | AGAGGCAACTGCAGACTCAT |
| **PR1_R**, 3208 | GTGTTCGCAGCGTAGTTGTA |
| **PR2_F**, 3346 | GTTTCTGGAGCAGGGCTTGA |
| **PR2_R**, 3347 | AGTAAGGGTAGAGATTCACGAGCA |
| **SNC1_F**, 7882 | AATCTCACTGGATGCCCGAAT |
| **SNC1_R**, 7883 | AAGGCATACATCTCGTAAGGCAG |
