# Supplementary material for: The Arabidopsis SUMO E3 ligase SIZ1 mediates the temperature dependent trade-off between plant immunity and growth
Source: PLoS Genet. 2018 Jan 22;14(1):e1007157. doi: 10.1371/journal.pgen.1007157 (PMC5794169; doi:10.1371/journal.pgen.1007157)
Supplement: S3 Table — (DOC) [file pgen.1007157.s003.doc]

**Supplementary table 3.**  Primer combinations used for genotyping the different alleles.

| **Allele** | **Primer combinations used, including the analysis method** |
| --- | --- |
| ***SIZ1*^WT^,** | 3362+3363, |
| ***siz1-2*** | 3362+3278. |
| ***PAD4*^WT^,** | PCR 1: 3367+3368, PCR 2: 3366+3367, followed by digestion with *Bfi*I.  WT allele is cleaved while the *pad4-1* allele is not. |
| ***pad4-1*** |
| ***EDS1*^WT^,** | 4138+4139: The *eds1-2* alleles give an amplicon of 0.4 kb, while the WT allele gives an amplicon of 1.4 kb. |
| ***eds1-2*** |
| ***SID2*^WT^,** | 3275+3276: The amplicon was analyzed by DNA sequencing. Nucleotide 1347 is a C for the WT allele, while a T for the *sid2-1* allele. |
| ***sid2*** |
| ***SNC1*^WT^,** | 3604+3605, |
| ***snc1-11*** | 3603+3604. |
| ***SGT1A*^WT^,** | 3788+3789, |
| ***sgt1a KO*** | 3788+3370. |
| ***SGT1B*^WT^,** | 3782+3783: The amplicon was analyzed by DNA sequencing. Nucleotide 2228 is a G for the WT allele, while a A for the *eta3* allele. |
| ***sgt1b eta3*** |
| ***RAR1*^WT^,** | 3786+3787: The amplicon was analyzed by DNA sequencing. Nucleotide n°964 is a C in WT allele, while a T for the *rar1-21* allele. |
| ***rar1-21*** |
