# Supplementary figures and images for: The Arabidopsis SUMO E3 ligase SIZ1 mediates the temperature dependent trade-off between plant immunity and growth
Source: PLoS Genet. 2018 Jan 22;14(1):e1007157. doi: 10.1371/journal.pgen.1007157 (PMC5794169; doi:10.1371/journal.pgen.1007157)

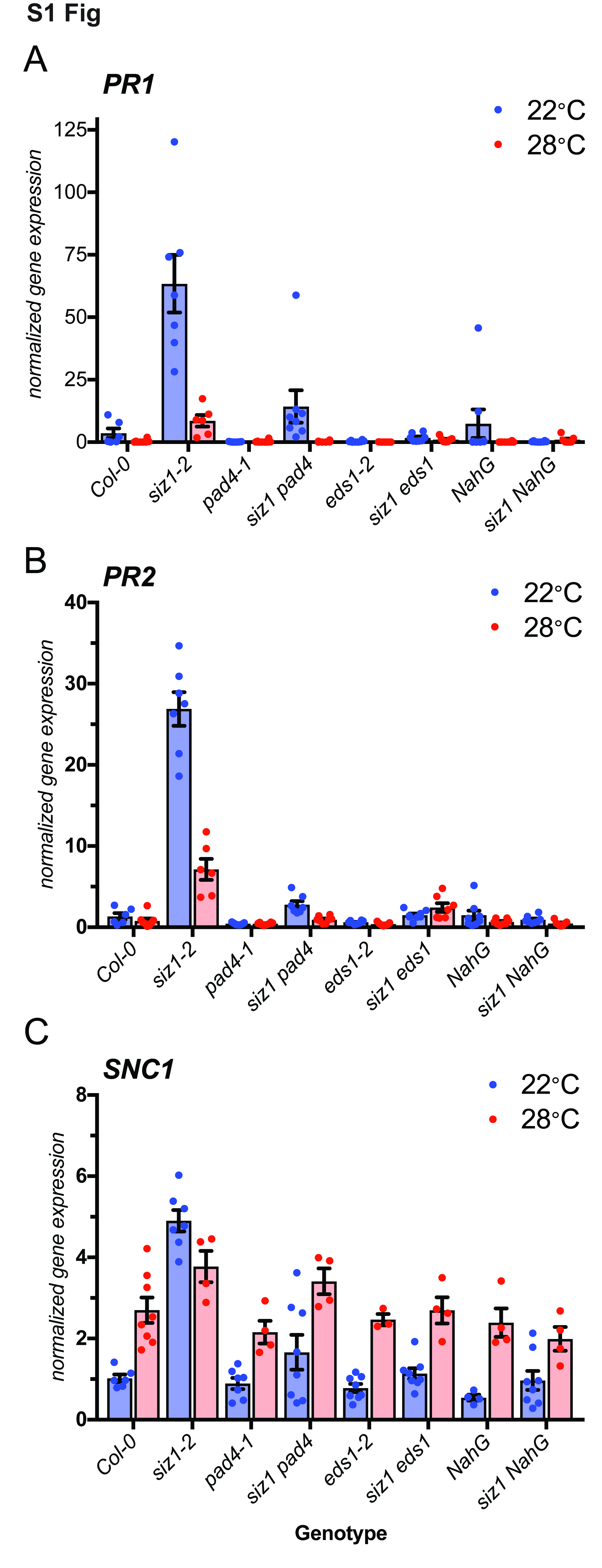

Supplement: S1 Fig — Normalized gene expression of the defence marker genes PR1 (A), PR2 (B) and SNC1 (C) using qRT-PCR (mean ± SE, Col-0 at 22°C = 1). RNA was isolated from 5-week-old plants. 3–4 biological replicates were measured in technical replicate. The experiment was repeated twice and the data combined. Experiment is part of the same set shown in Fig 2E. (JPG) [file pgen.1007157.s004.jpg]

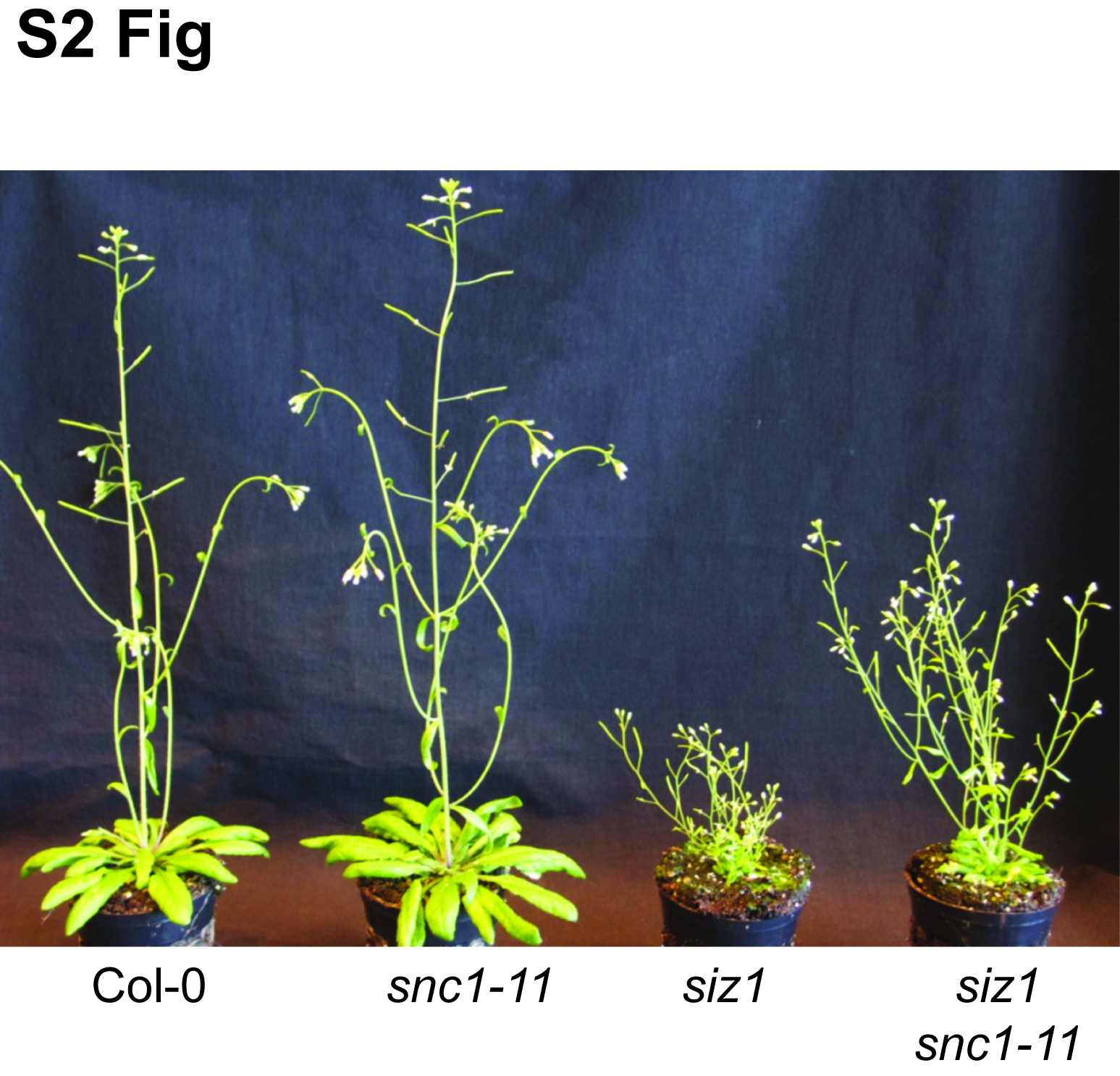

Supplement: S2 Fig — The picture was taken using 6-week-old flowering plants. (JPG) [file pgen.1007157.s005.jpg]

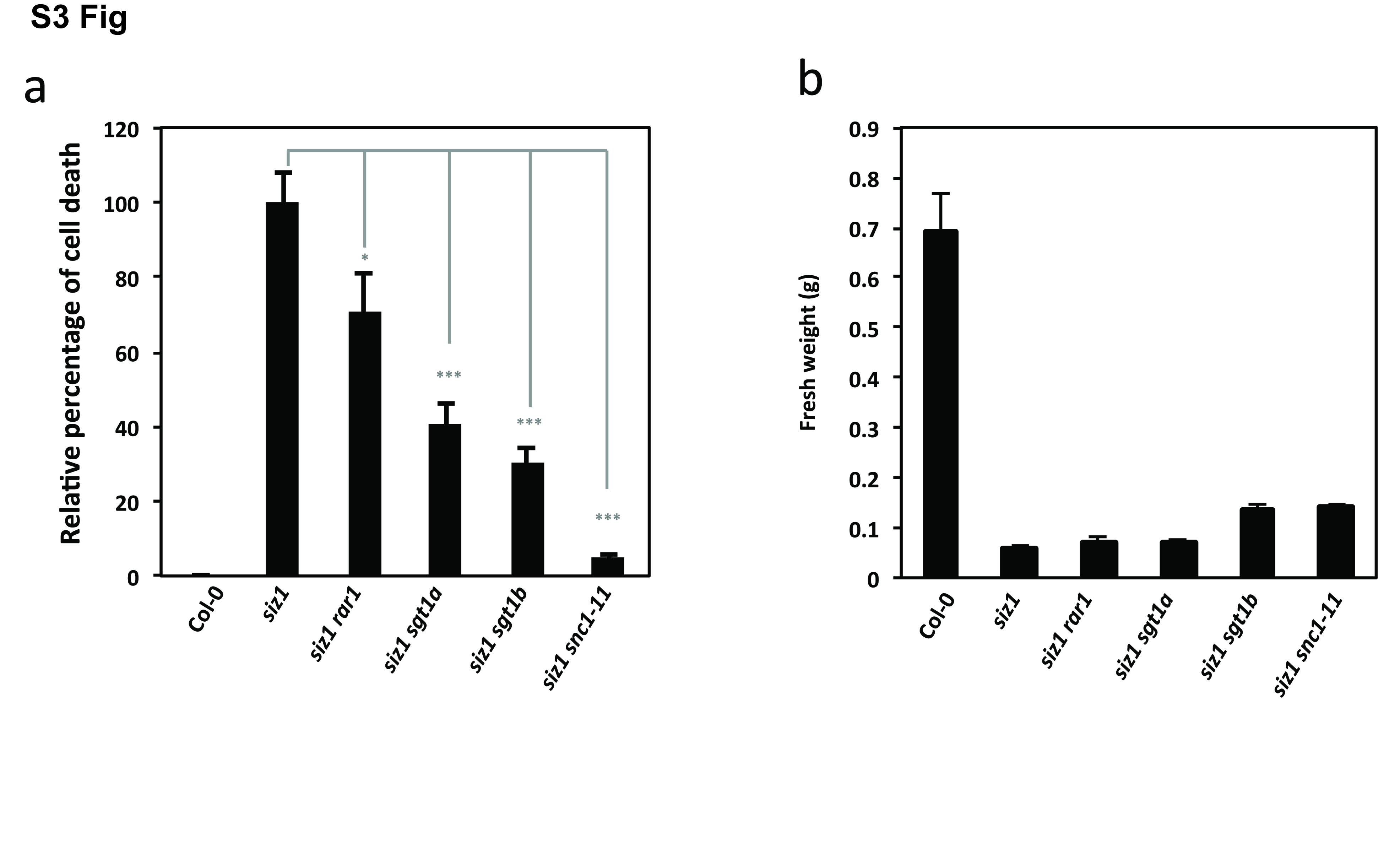

Supplement: S3 Fig — (A) Loss-of-function mutants of RAR1, SGT1a and SGT1b were introduced in siz1-2 by crossing. The double mutants show less cell death than the siz1 single mutant. siz1 snc1-11 is included as neg. control (see Fig 2C). Leaves of 5-week-old plants were stained with Trypan blue. To quantify cell death the number of lesions was counted per leaf size area for each genotype. At least 10 images were counted per genotype. Statistical analyses were made using an unpaired two-sided student t-test (grey lines) with ns for p>0.05; * for p≤0.05; ** for p≤0.01 and *** for p≤0.001. (B) Introduction of loss-of-function mutants of RAR1, SGT1a and SGT1b in siz1 hardly rescues the growth retardation of siz1. Rosette weight was taken from 5-week-old plants (n = 8). (JPG) [file pgen.1007157.s006.jpg]

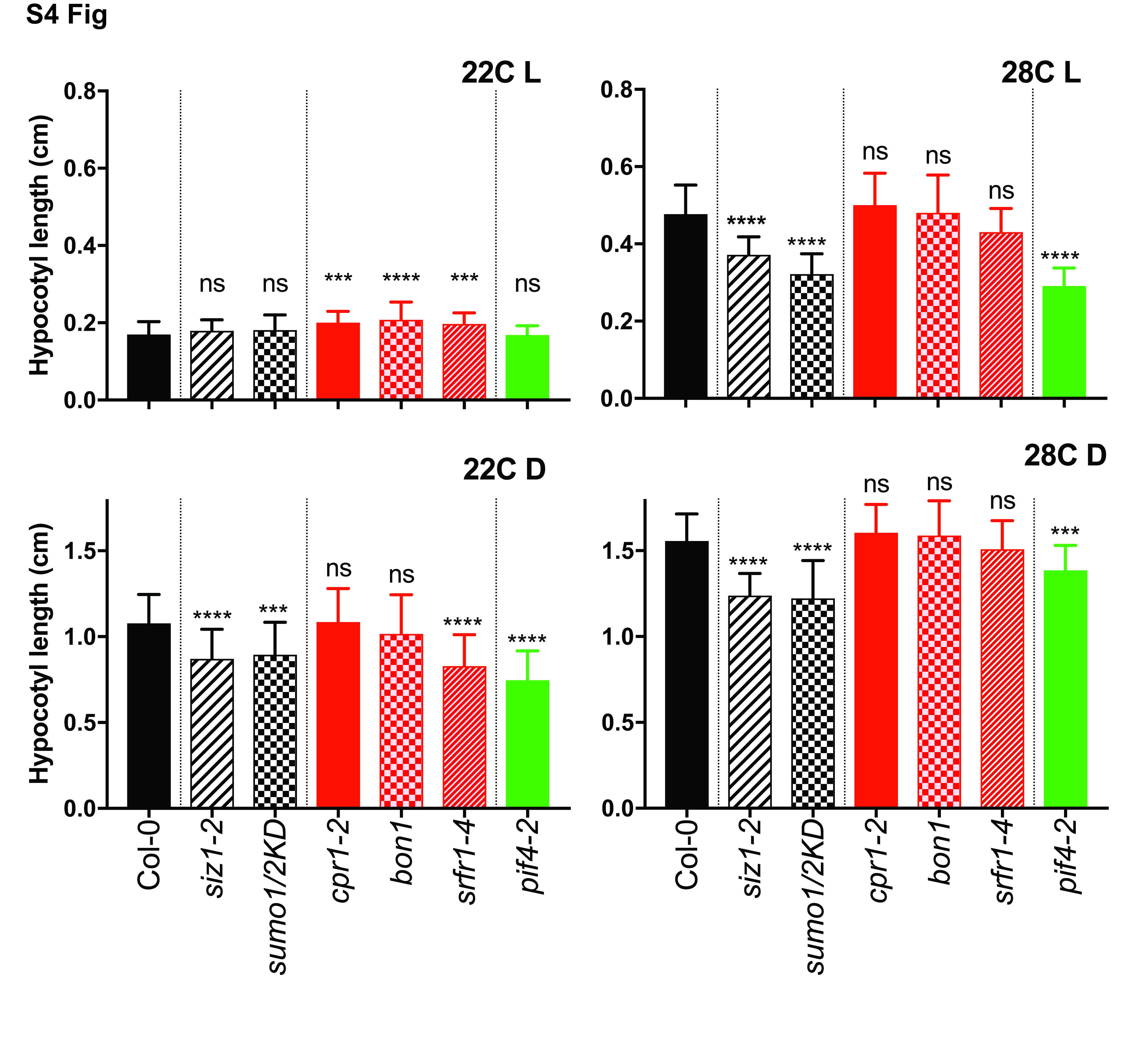

Supplement: S4 Fig — Whereas hypocotyl growth is compromised in siz1 and sumo1/2KD, the mutants cpr1, bon1, and srfr1-4 show normal hypocotyl elongation at elevated temperature (both in a diurnal cycle and in dark conditions; 28C L and 28C D, respectively). Only srfr1-4 shows less hypocotyl elongation in dark conditions at 22°C (28C D). Seeds were germinated on plates at 22°C/28°C in SD (L) or dark (D) conditions. Hypocotyl length was measured 5 days post germination. Significant differences were determined using ANOVA followed by Tukey post-hoc test (****, p≤0.0001; ***, p≤0.001; ns, p>0.05; n = 40–43). All significant differences indicated are in comparison to Col-0 (control). The result shown was part of the experiment in Fig 4A and 4B. Experiment was repeated two times with similar results. Error bars indicate standard deviation. (JPG) [file pgen.1007157.s007.jpg]

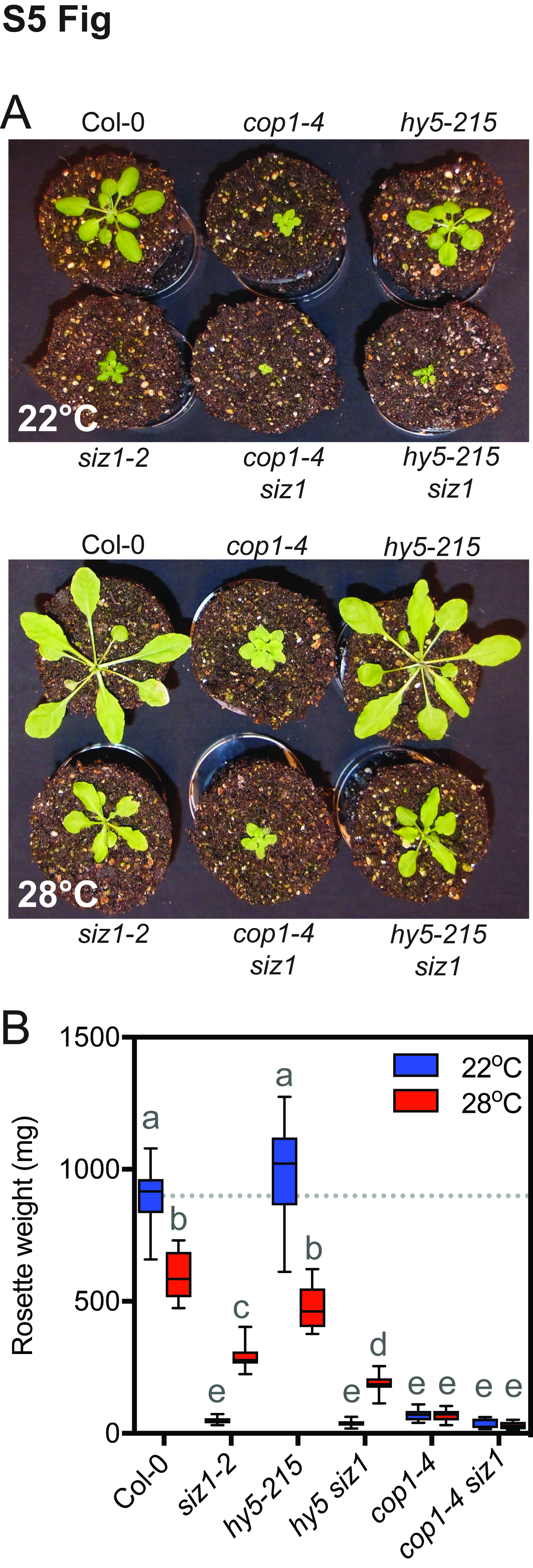

Supplement: S5 Fig — (A) Picture of the rosettes of siz1 hy5-215, siz1 cop1-4 and the single mutants. Plants were grown for 5 weeks at 22°C or 28°C (SD). The double mutants adopted a similar morphology as siz1-2 at 22°C. while the growth retardation of siz1 hy5-215 partially recovered at 28°C albeit slightly less than siz1 alone. The rosette of siz1 cop1-4 remained as compact as cop1-4 alone without petiole elongation at 28°C, indicative of a compromised thermomorphogenesis response. (B) Box-plot (middle bar = median, box limit = upper and lower quartile, extremes = Min and Max values) showing the rosette weight of the genotypes depicted in (A). Weight was taken from 5-week-old plants. Significant differences were detected using a two-way ANOVA with Tukey’s multiple comparisons test; the letters indicate significantly different groups (n = 8–10). The experiment was repeated twice times with similar result. (JPG) [file pgen.1007157.s008.jpg]

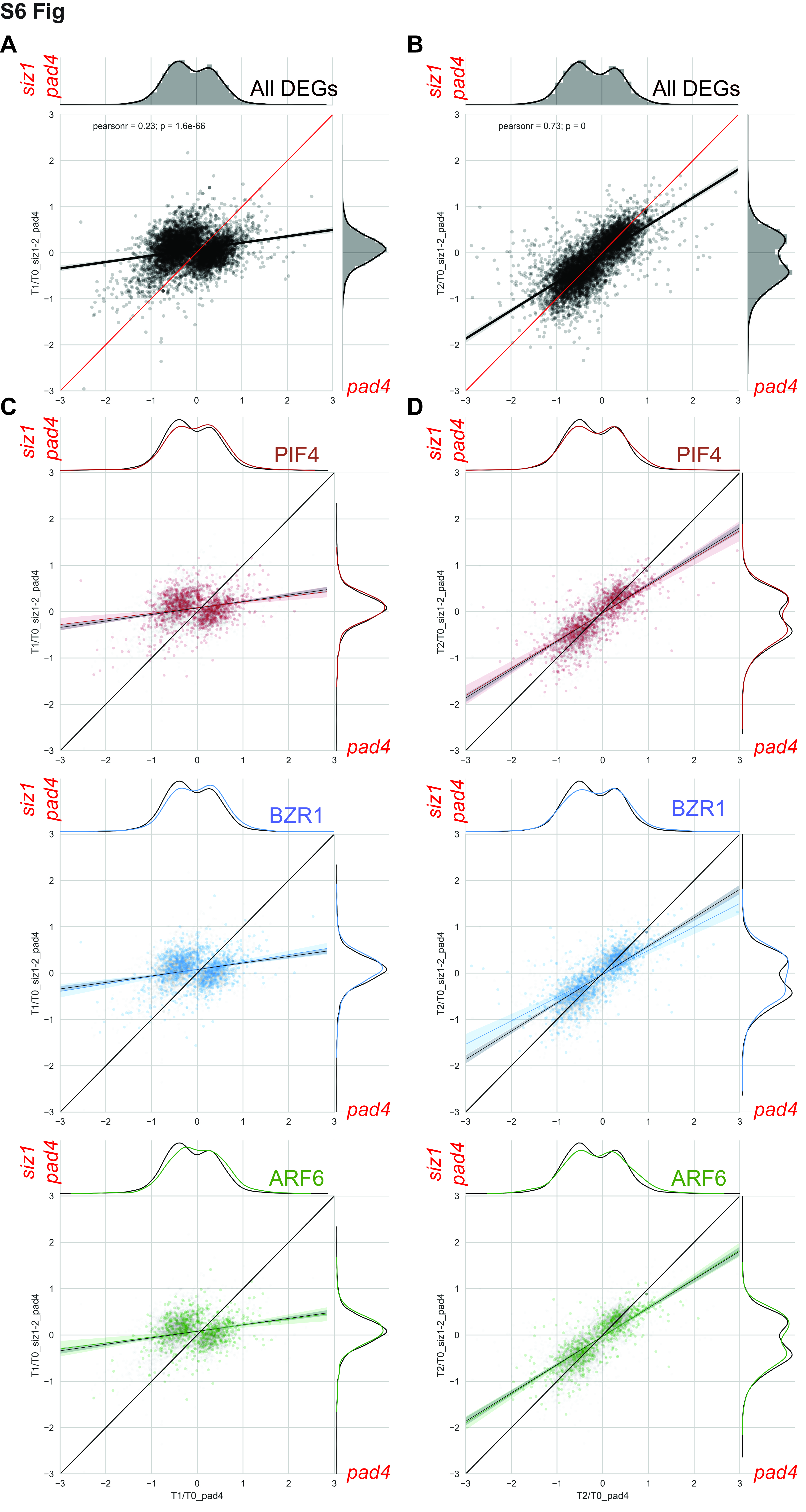

Supplement: S6 Fig — (A, B) Scatter plot showing the log2 fold change in expression of all DEGs (black spots) at the three time points in siz1 pad4 versus pad4 (identical to Fig 5C and 5D) for [day 1–0] and [day 4–0], respectively. The black line depicts a Pearson linear regression result on the DEGs with the 95% confidence interval indicated by the grey zone. (C, D) Similar to (A, B) except that only the DEGs are shown that are also genomic targets for binding of PIF4 (red spots), BZR1 (blue spots) or ARF6 (green spots), top-to-bottom. The red, blue and green lines depict the Pearson linear regression analysis on these DEGs that are also genomic targets of these different TFs with the 95% confidence interval indicated by the red, blue or green zone. (JPG) [file pgen.1007157.s009.jpg]

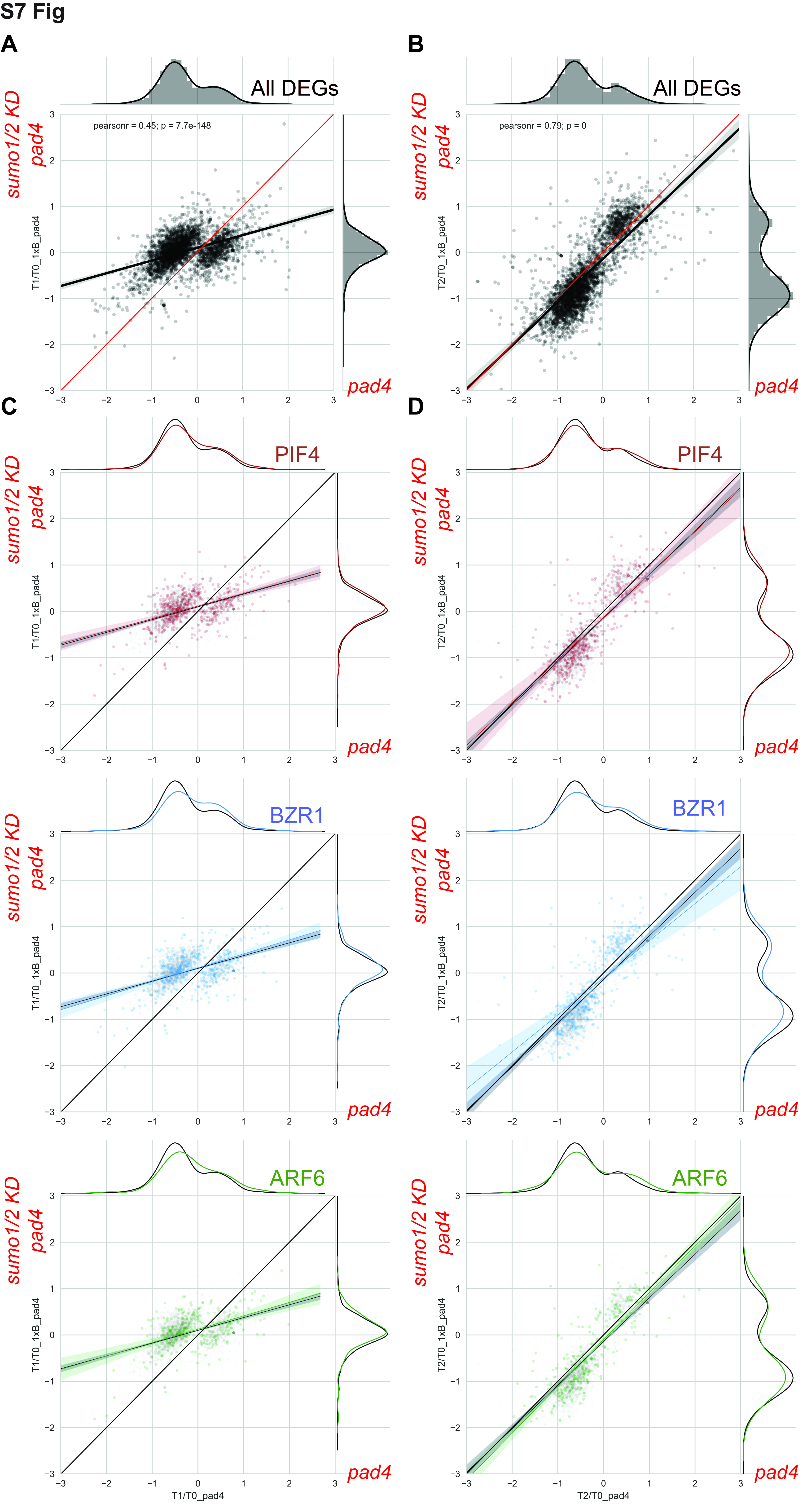

Supplement: S7 Fig — (A, B) Scatter plot showing the log2 fold change in expression of all DEGs (black spots) at the three time points in sumo1/2KD pad4 versus pad4 (identical to Fig 5C and 5D) for [day 1–0] and [day 4–0], respectively. The black lines depict a linear Pearson regression analysis on the DEGs with the 95% confidence interval indicated by the grey zone. (C, D) Similar to (A, B) except that only the DEGs are shown that are also genomic targets for binding of PIF4 (red spots), BZR1 (blue spots) or ARF6 (green spots), top-to-bottom. The red, blue and green lines depict a Pearson linear regression analysis on these DEGs that are also genomic targets of PIF4, BZR1 or ARF6, respectively, with the 95% confidence interval indicated by the red, blue or green zone. (JPG) [file pgen.1007157.s010.jpg]

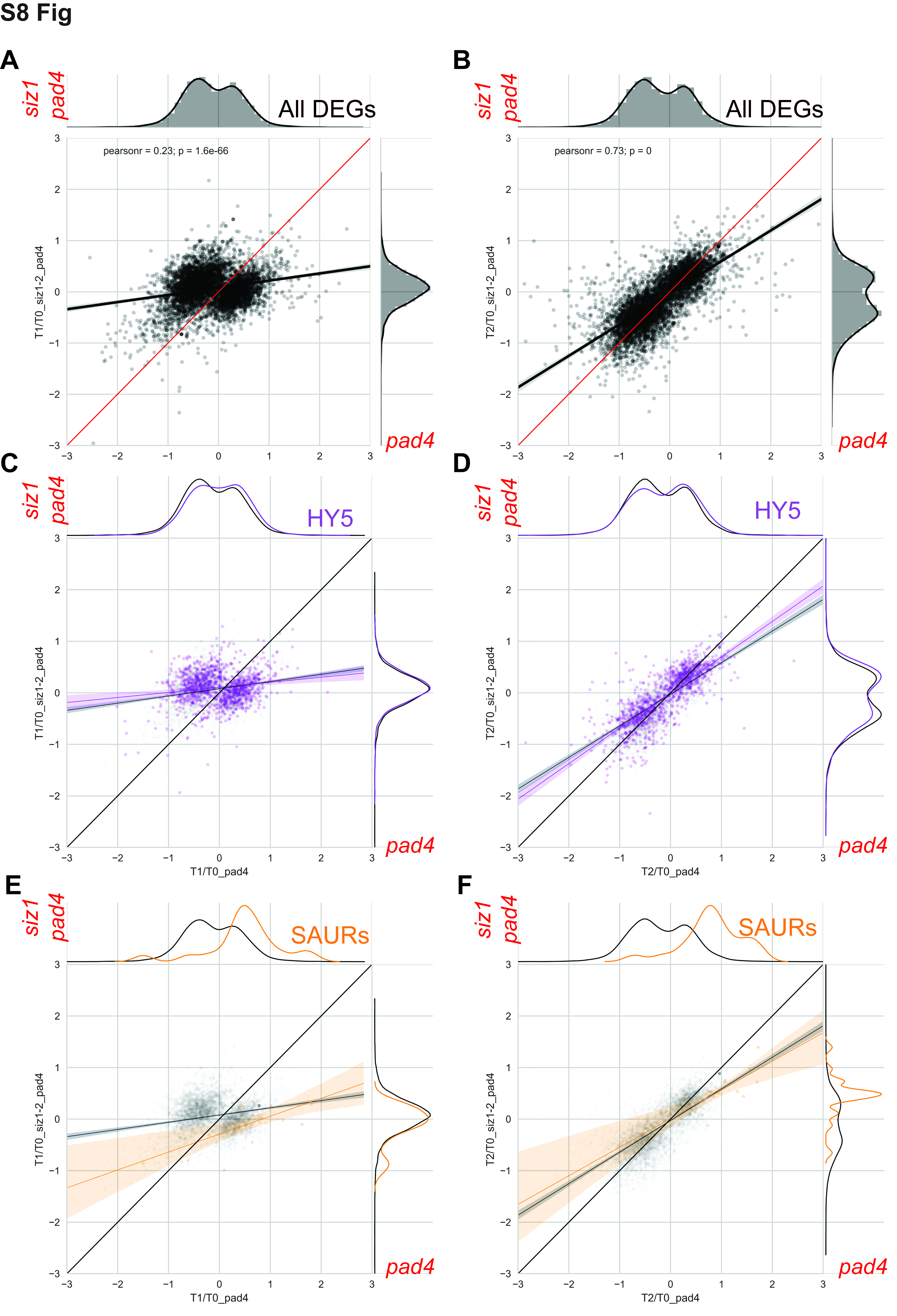

Supplement: S8 Fig — (A, B) Scatter plot showing the log2 fold change in expression of all DEGs (black spots) at the three time points in siz1 pad4 versus pad4 (identical to Fig 5C and 5D) for [day 1–0] and [day 4–0], respectively. The black lines depict a Pearson linear regression analysis of the differentially expressed genes with the 95% confidence interval indicated by the grey zone. (C, D) Similar to (A, B) except that only the DEGs are shown that are also a genomic target for binding of HY5 (purple spots). The purple line depicts a Pearson linear regression analysis on these DEGs that are also genomic targets of HY5, with the 95% confidence interval indicated by the purple zone. (E, F) Similar to (A, B) except that only the DEGs are shown that encode for SAUR genes (yellow spots). These SAUR genes are clearly up-regulated in pad4 at day 1 and 4, but they fail to respond at day 1 and their response is irregular at 4 day in siz pad4. The yellow line depicts a Pearson linear regression analysis on these DEGs that encode SAURs with the 95% confidence interval indicated by the yellow zone. (JPG) [file pgen.1007157.s011.jpg]

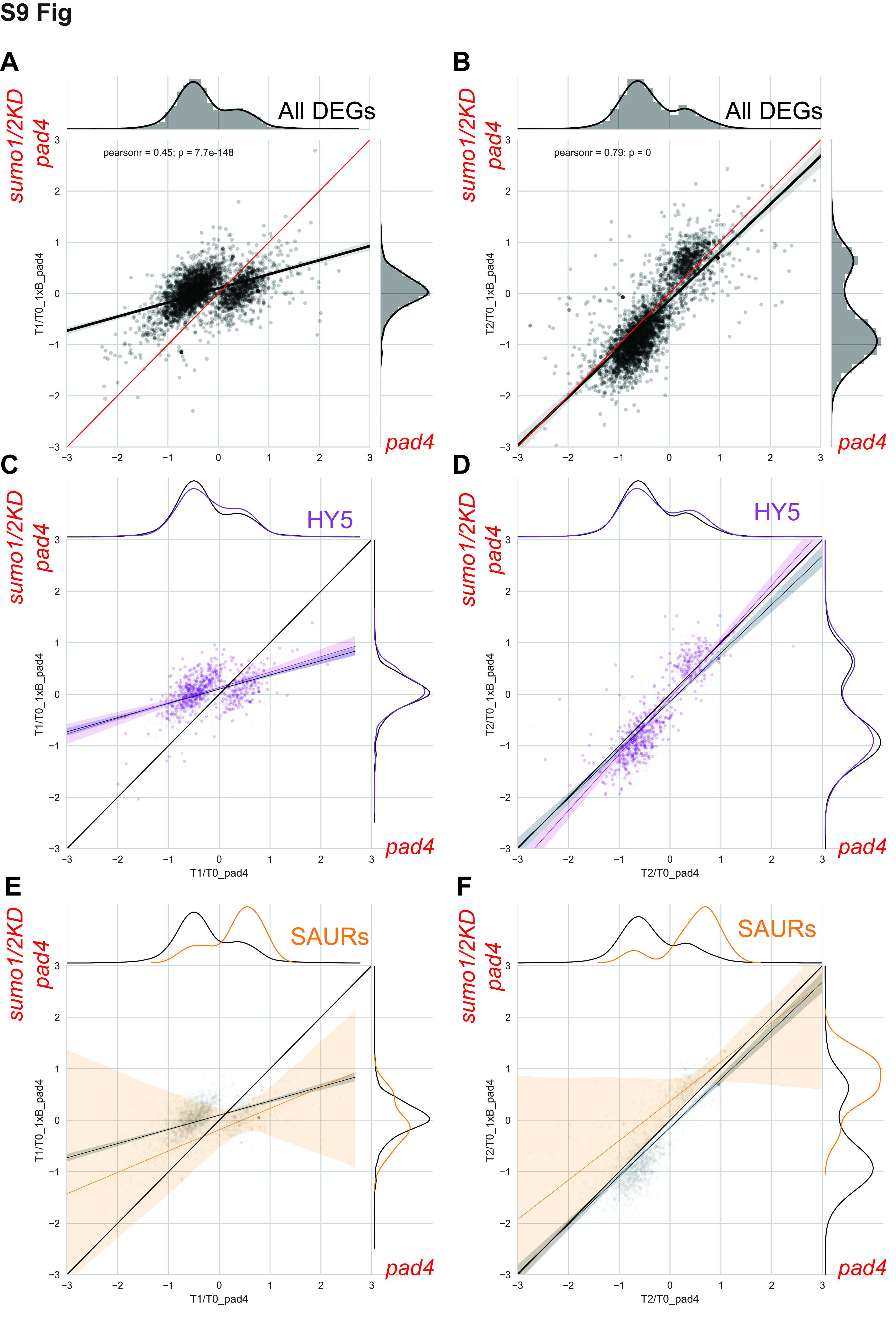

Supplement: S9 Fig — (A, B) Scatter plot showing the log2 fold change in expression of all DEGs (black spots) at the three time points in sumo1/2KD pad4 versus pad4 (identical to Fig 5C and 5D) for [day 1–0] and [day 4–0], respectively. The black lines depict a Pearson linear regression analysis on the differentially expressed genes with the 95% confidence interval indicated by the grey zone. (C, D) Similar to (A, B) except that only the DEGs are shown that are also a genomic target for binding of HY5 (purple spots). The purple line depicts a Pearson linear regression analysis on the DEGs that are also genomic targets of HY5, with the 95% confidence interval indicated by the purple zone. (E, F) Similar to (A, B) except that only the DEGs are shown that encode for SAUR genes (yellow spots). The yellow line depicts a Pearson linear regression analysis on these DEGs that encode SAURs with the 95% confidence interval indicated by the yellow zone. (JPG) [file pgen.1007157.s012.jpg]
